# Molecular characterization of ferroptosis in soft tissue sarcoma constructs a prognostic and immunotherapeutic signature through experimental and bioinformatics analyses

**DOI:** 10.18632/aging.205133

**Published:** 2023-10-20

**Authors:** Zhi-Qiang Yang, Liang-Yu Guo, Kang-Wen Xiao, Chong Zhang, Min-Hao Wu, Fei-Fei Yan, Lin Cai

**Affiliations:** 1 Department of Orthopedics, Zhongnan Hospital of Wuhan University, Wuhan, Hubei 430071, People’s Republic of China; 2 Department of Orthopedics, Renmin Hospital of Wuhan University, Wuhan, Hubei 430071, People’s Republic of China; 3 School of Medicine, Washington University, St. Louis, MO 63110, USA

**Keywords:** ferroptosis, soft tissue sarcoma, immunotherapy, nomogram, survival analysis

## Abstract

Ferroptosis regulators have been found to affect tumor progression. However, studies focusing on ferroptosis and soft tissue sarcoma (STS) are rare. Somatic mutation, copy number variation, reverse transcription-quantitative polymerase chain reaction (RT-qPCR) analysis, consensus clustering, differentially expressed genes analysis (DEGs), principal component analysis (PCA) and gene set enrichment analysis (GSEA) were used to identify and explore different ferroptosis modifications in STS. A nomogram was constructed to predict the prognosis of STS. Moreover, three immunotherapy datasets were used to assess the Fescore. Western blotting, siRNA transfection, EdU assay and reactive oxygen species (ROS) measurement were performed. 16 prognostic ferroptosis regulators were screened and significant differences were observed in somatic mutation, copy number variation (CNV) and RT-qPCR among these ferroptosis regulators. 2 different ferroptosis modification patterns were found (Fe cluster A and B). Fe cluster A with higher Fescore was correlated with p53 pathway and had better prognosis of STS (*p* = 0.002) while Fe cluster B with lower Fescore was correlated with angiogenesis and MYC pathway and showed a poorer outcome. Besides, the nomogram effectively predicted the outcome of STS and the Fescore could also well predict the prognosis of other 16 tumors and immunotherapy response. Downregulation of LOX also inhibited growth and increased ROS production in sarcoma cells. The molecular characterization of ferroptosis regulators in STS was explored and an Fescore was constructed. The Fescore quantified ferroptosis modification in STS patients and effectively predicted the prognosis of a variety of tumors, providing novel insights for precision medicine.

## INTRODUCTION

With multiple subtypes and different clinical outcomes, STS has attracted researchers’ attention in the recent years [1]. Being a rare tumor, STS accounted for 0.71% of estimated new cases based on the latest cancer statistics in United States [2]. According to different origin and morphology, STS can be also divided into liposarcoma, hemangioma, neuroma, etc. At present, the main treatment of soft tissue sarcoma is surgery resection combined with radiology therapy, which reduces the risk of reoccurrence [3]. However, the probability of STS patients being alive at 5 years is only 10% [4]. Therefore, exploring new targets and implementing individualized treatment is of great significance to improve the prognosis of STS patients.

Ferroptosis is characterized by the iron dependent accumulation of lipid peroxidation to a lethal level [5]. Recent studies have revealed a close relationship between ferroptosis and tumor progression. Ferroptosis regulator HSPB1 was also related to decreased ferroptosis by reducing the expression of TFR1, which further accelerated cervical carcinoma growth [6]. Another research revealed that rhabdomyosarcoma cells death was induced by Erastin through lipid peroxidation and reactive oxygen species production, which were typical features of ferroptosis [7]. Besides, recent research showed that ME1 absence could lead to synovial sarcoma cells being more sensitive to ferroptotic cell death [8]. Moreover, desmoid-type fibromatosis cells death could be induced by sorafenib through ferroptosis [9]. Considering the close relationship between ferroptosis and the progression of STS and other tumors, it is very necessary to further explore the ferroptosis modification patterns in STS.

Since the advent of immunotherapy, it has been of wide interest to clinicians and scientists all over the world. Immunotherapy mainly included PD-1/PD-L1 and CTLA-4, which were also called immune checkpoint blockade (ICB) [10]. Although ICB has made good achievements against cancer, only a small number of patients could respond to ICB, and the price of treatment was expensive, which greatly limited the use of ICB [11]. Tumor microenvironment (TME); microenvironment around tumor cells, also plays an important role in regulating the outcome of immunotherapy. A recent study reported that PD-L1 could be regulated by Interferon γ in vascular endothelial cell [12]. Besides, interleukin-17 (IL-17) in the TME could also increase the expression of PD-L1 in prostate cancer and colon cancer, which further affected immunotherapy response [13]. A previous study also revealed that transforming growth factor beta (TGF-β) could enhance the expression of PD-1/L1 in hepatocellular carcinoma [14]. Although the relationship between TME and tumor immunotherapy have been discussed, these studies were not associated with ferroptosis. Hence, it was imperative to explore the modification pattern of ferroptosis in STS and identify novel predictive markers for immunotherapy response.

Recently we explored the role of N6-methyladenosine modification in STS [15]. Based on this experience, here 16 prognostic ferroptosis regulators were screened through univariate Cox regression analysis. RT-qPCR, somatic mutation and copy number variation further revealed significant differences of these regulators in STS. Two ferroptosis modification patterns were identified using consensus clustering [16]. Moreover, significant differences in immune infiltration and prognosis between the ferroptosis modification patterns were observed by CIBERSORT, ESTIMATE and survival packages [17]. Hence, we constructed an Ferroptosis-related score (Fescore), which well predicted the immune infiltration and prognosis of STS. The Fescore could also predict the response to ICB, which provided new insights for precision medicine and targeted therapy.

## MATERIALS AND METHODS

### Sample collection and data processing

In this study, 33 types of cancer and the corresponding survival information in The Cancer Genome Atlas (TCGA) were downloaded from UCSC-XENA (http://xena.ucsc.edu/). The somatic mutation and CNV of TCGA-SARC (*n* = 265) were also obtained from UCSC-XENA. Sarcoma datasets GSE17674 [18], undifferentiated sarcoma (GSE119041 [19]), Liposarcoma (GSE159848 [20]), Leiomyosarcoma (GSE159847 [20]), Synovial Sarcoma (GSE40021 [21]), Ewing Sarcoma (GSE17168 [18]), and Fibrosis sarcoma (GSE71118 [22]) were collected from Gene Expression Omnibus (GEO) database. GPL570 platform was used for GSE17674, GSE17618 and GSE71118 while gencode platform (https://www.gencodegenes.org/) was used for 33 types of cancer in TGCA. GPL6480 platform was used for GSE159848, GSE40021 and GSE159847. GPL17692 platform was used for GSE119041. Robust multi-array average (RMA) normalization was applied in GSE17674 and GSE17618 and Transcripts Per Kilobase Million (TPM) normalization was performed in 33 types of cancer. GCRMA algorithm was applied in GSE71118 and Quantile algorithm was applied in GSE159848, GSE40021 and GSE159847. Data from GSE119041 were normalized with the default settings on Thermo Fishers Expression Console 1.4.1.46 Software.

**Figure 1:**
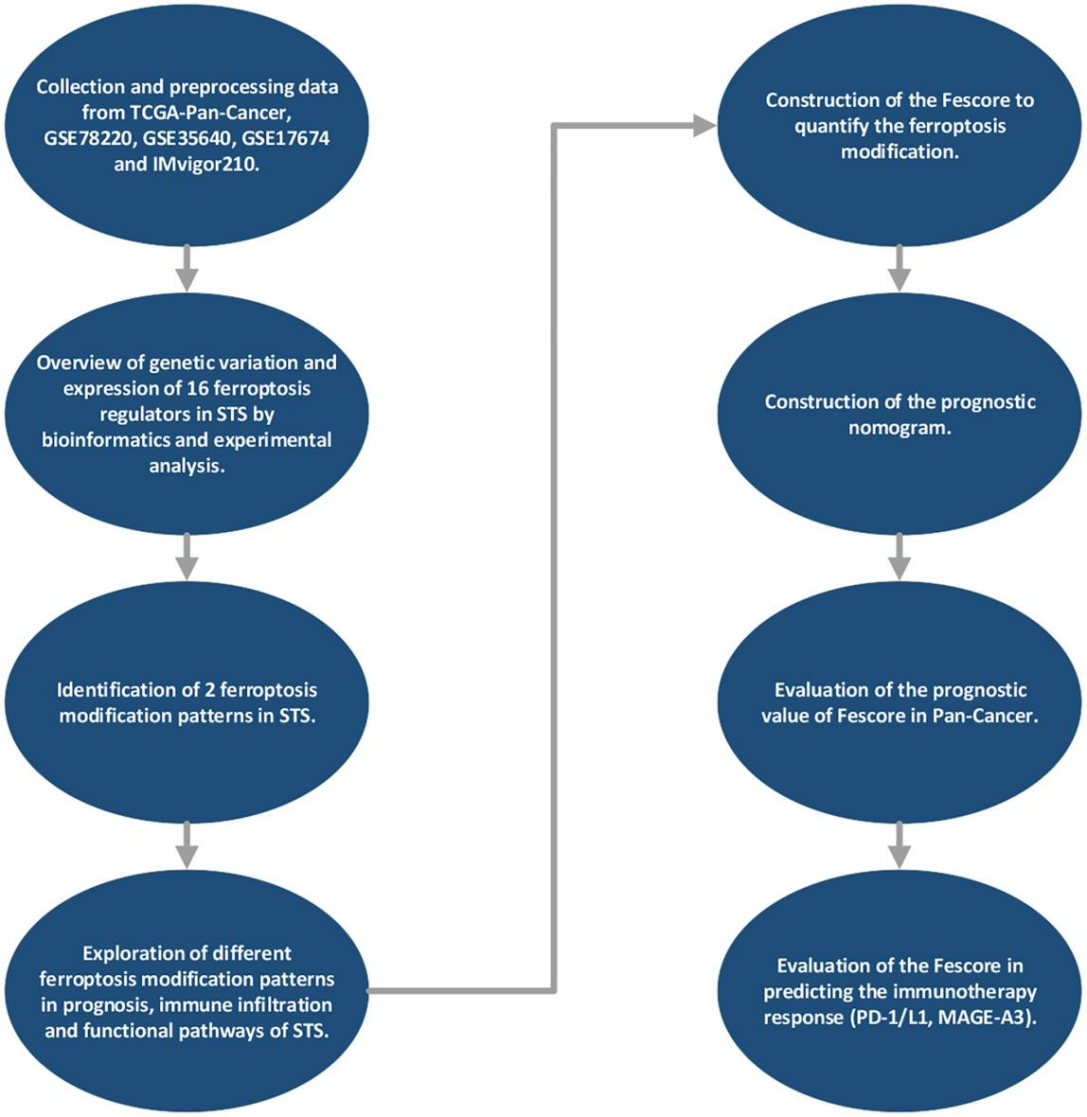
The flowchart of this study.

Three immunotherapy datasets IMvigor210 [18] (*n* = 298), GSE78220 (*n* = 28) and GSE35640 (*n* = 65) were collected from recent studies. IMvigor210, GSE78220 and GSE35640 were normalized by trimmed mean of M-values, FPKM (Fragments Per Kilobase Million) and RMA, respectively. Ferroptosis regulators were screened according to recent research [23]. Samples without full clinical characteristics were excluded from this study. Three STS samples and the corresponding normal tissue were obtained from Department of Orthopedics, Zhongnan Hospital of Wuhan University. Detailed information of these datasets was shown in Supplementary Table 1. The list of ferroptosis regulators was shown in Supplementary Table 2. The flowchart was shown in Figure 1.

### LOX knockdown using siRNA transfection

A siRNA strategy was used to knock down LOX in HT1080 and A204 cells. Briefly, 1 × 10^5^ cells were added to fresh medium without antibiotics and seeded in six-well plates 24 hrs before transfection. For transfection, all siRNAs (si-LOX or the negative control, siNC) (Tsingke Biotechnology Co., Ltd., Beijing, China) were resuspended at a concentration of 20 μM and then transfected into HT1080 and A204 cells. TSnanofect V1 transfection reagent (Tsingke Biotechnology) was used when the cells reached 50–70% confluence. A transfection rate of 70–85% of cells was used for further experiments. The siRNA sequences targeting LOX were shown in Supplementary Table 3.

### Western blotting

HT1080 and A204 cells were seeded in 6-well plates, and then transfected with NC group and Si group respectively for 48 hours and then collected. Cells were washed once with PBS and then lysed with Protein Lysis Buffer containing protease and phosphatase inhibitor cocktail for 30 min on ice. Cell lysates were centrifuged at 12,000 × g for 15 min at 4°C, and the supernatant was collected. Protein concentration was quantified using the BSA protein assay according to the manufacturer’s instructions. Equal amounts of total protein were separated by SDS-PAGE (8–12%) at 80–120 V for 1.5 h and transferred to a 0.45 μM PVDF membrane at 230 mA for 1.5 h. After blocking with 5% skim milk in TBST buffer for 1 h at room temperature, membranes were incubated with primary antibodies anti-GAPDH and ant-LOX (GAPDH, LOX antibodies were purchased from ProteinTech (Wuhan, China)) overnight at 4°C. Membranes were washed three times with TBST buffer and then incubated with peroxidase-conjugated secondary antibodies for 1 h at room temperature. Specific antibody binding was detected by a chemiluminescence kit.

### EdU assay

EdU assay was performed using the EdU–555 Cell Proliferation Assay Kit (Beyotime, Shanghai, China). Observation and photography were performed using a fluorescence microscope (Olympus, Tokyo, Japan).

### ROS

To assess the intracellular ROS scavenging activity, the cells were stained with the fluorescent 2,7-dichlorodihydrofluorescein diacetate (DCFH-DA) probe with ROS assay kit as previously described [24]. In short, HT1080 and A204 cells were harvested and collected 48 hours after transfection, and washed with serum free medium. Then, the cells were combined with 10 μM DCFH-DA probes (Molecular Probes, Eugene, OR, USA) and were mixed in serum-free medium and incubated in 37°C darkness for 30 minutes, with slight stirring every 5 minutes. Then, we collected the cell granules, washed them with PBS three times, and BS is used for flow cytometry analysis. Induced green fluorescent nanoparticles from 10000 cells were recorded at 488 nm. FlowJ software was used to analyze the average fluorescence intensity.

### Transwell assays

A Transwell assay was performed using a Transwell system (24 wells, 8 μm pore size with polycarbonate membrane) according to the manufacturer’s instructions. First, HT1080 and A204 cells were seeded into the upper chamber with serum-free corresponding medium. Medium with 10% FBS was put into the lower compartment, and the cells were allowed to migrate for 48 h. The remaining cells in the upper chamber were scraped out by a cotton swap. Cells were fixed with ice-cold methanol and stained with 0.1% crystal violet solution. The number of cells that migrated to the lower side was counted in five randomly selected fields under a light microscope. The cell number was counted and analyzed statistically.

### Cell line, RNA extraction and RT-qPCR

Human skeletal muscle cell line (HSMC) and sarcoma cell line (A673) were purchased from the American Type Culture Collection (Manassas, VA, USA). Human fibrosarcoma cell line HT1080 and rhabdomyosarcoma cell line A204 were purchased from Procell (Wuhan, China). A673 and HSMC were cultured in RPMI 1640 medium (Hyclone, Logan, UT, USA) and DMEM (Hyclone) respectively. HT-1080 and A204 were cultured in MEM (Procell) and McCoy’s 5A medium (Procell). All complete media were supplemented with 10% fetal bovine serum (Gibco, Langley, OK, USA) and 1% antibiotics (100 U/mL penicillin, 100 μg/mL streptomycin). The cells were maintained in an incubator with 37°C and 5% CO2. The total RNA of cell lines and tissue was extracted by Trizol method (Invitrogen, Waltham, MA, USA), and then the RNA was reverse transcribed by reverse transcription kit (Roche, Basel, Switzerland) to obtain cDNA; RT-qPCR was performed according to the instructions. The prime sequences of all genes were listed in Supplementary Table 4.

### Identification of different ferroptosis modification patterns in STS through consensus clustering analysis

Prognostic ferroptosis regulators were screened using survival and survminer packages [25]. Then different ferroptosis modification patterns were calculated through consensus clustering analysis according to the expression of these 16 prognostic ferroptosis regulators. The above analysis was performed 1000 times to achieve stable clustering by ConsensusClusterPlus package.

### Differentially expressed genes analysis and connectivity map analysis

DEGs were performed between different ferroptosis modification patterns by limma package [26]. Benjamini-Hochberg procedure was performed to adjust multiple hypothesis [27]. The requirement for DEGs was: adjust *p* < 0.050 and log^FC^ > 1 or log^FC^ < −1. Connectivity Map (cMap) was applied to reveal the functional relationship between small molecule compounds, genes and disease status [28]. The above DEGs were subsequently used for cMap analysis to explore potential drugs for STS. *p* < 0.05 indicated statistical significance.

### Immune cell infiltration in STS

The CIBERSORT algorithm was used to explore the immune infiltration in STS samples. LM22 signature was used here and the permutation was set to 1000. Then ESTIMATE package was further applied to calculate the STS purity score [29].

### Functional enrichment analysis

The 16 prognostic ferroptosis regulators were used for Gene Ontology (GO) analysis by clusterProfiler package [30]. Significant pathways between different ferroptosis modification patterns were also identified using gene set enrichment analysis (GSEA) [31]. False discovery rate (FDR) < 0.05 was considered significant.

### Construction of the Fescore and prognostic nomogram

To quantify the ferroptosis modification patterns in STS, an Fescore was constructed according to previous experience [32]. Detailed process of calculating Fescore was as follows: First, the DEGs between different ferroptosis modification patterns were used for consensus clustering analysis. Then Cox regression was performed to screen prognostic DEGs. Finally, PCA was performed to establish the Fescore on the basis of prognostic DEGs after z-score standardization and principal component 1 was regarded as signature score. The formula of Fescore is shown below:


$$Fescore=\sum pc1_{m}-\sum pc1_{n}$$

m was prognostic DEGs with Hazard Ratio (HR) < 1 while n was prognostic DEGs with HR > 1.

Besides, the prognostic nomogram to predict the survival of STS was constructed based on gender, age, race, metastatic status, margin status and Fescore by rms package (https://hbiostat.org/R/rms/). Bootstrap method was used with 1000 iterations and the calibration curves of survival of STS were also used to assess the predictive function of the nomogram.

### Statistical analysis

Statistical Product and Service Solutions software (SPSS 22.0) and R 3.6.2 were used for data analysis. The somatic mutation of STS was displayed using maftool package [33]. The relative location of 16 prognostic ferroptosis regulators in human chromosome was shown by Rcircos package [34]. The relationship between different ferroptosis regulators and different immune cells were displayed by corrplot package, respectively [35]. Cox regression analysis [36] was used to explore prognostic ferroptosis regulators, prognostic DEGs and evaluate the prognostic value in other 32 types of cancer in TCGA. Survival package and survminer package were used for survival analysis and setting cut-off point. The heatmaps were drawn by Pheatmap package [37]. Log-rank test was used to compare the survival rate. The receiver operating characteristic (ROC) curve for predicting the prognosis of STS and immune response were performed by using timeROC package and pROC package, respectively. FactoMineR package [38] was used for PCA. Protein-protein interaction analysis was performed and visualized by STRING and cytoscape [39], respectively and hub genes were identified based on degree > 4. Kruskal-Walls test was used to compare differences among different clusters and groups. *p* < 0.05 was considered significant.

## RESULTS

### Overview of prognostic ferroptosis regulators variation in STS

**Figure 2:**
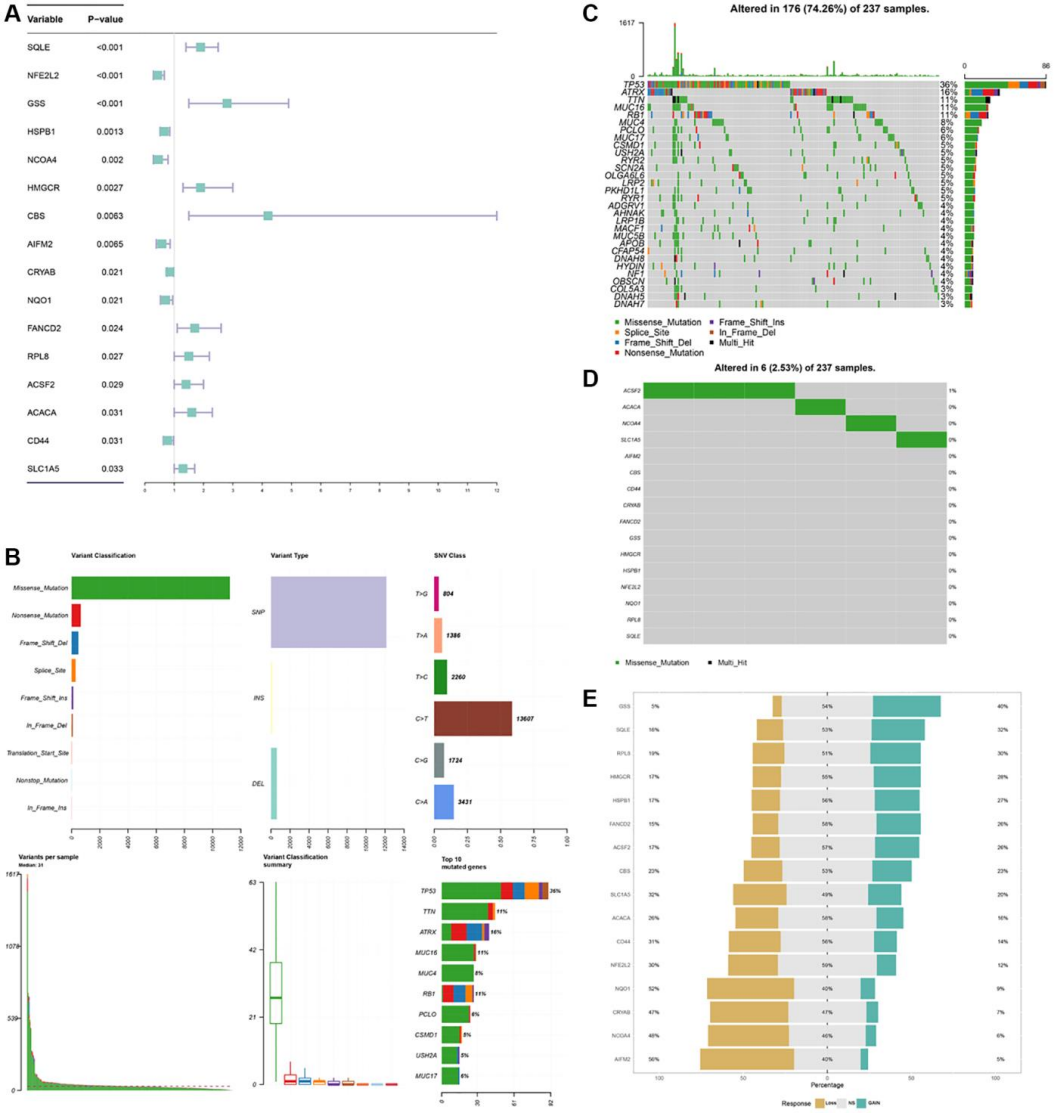
**Landscape of prognostic difference, somatic mutations, CNV of ferroptosis regulators in STS.** (**A**) Univariate Cox regression analysis for each ferroptosis regulators in STS. (**B**) Variant classifications of mutations in STS. (**C**) Summary of somatic mutations in STS. (**D**) Summary of somatic mutations of ferroptosis regulators in STS. (**E**) CNV of 16 ferroptosis regulators in STS.

**Figure 3:**
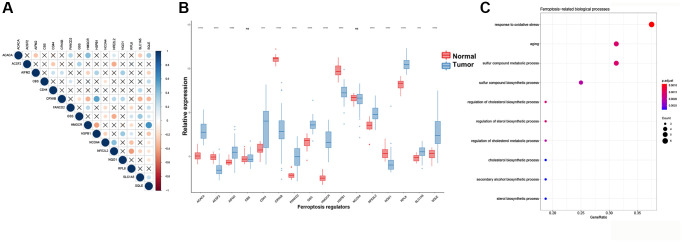
**Expression, interactions and functional annotations of ferroptosis regulators in STS.** (**A**) Correlation plot among 16 regulators using Pearson correlation analysis. PPI analysis of ferroptosis regulators. (**B**) Expression of different ferroptosis regulators between normal samples and STS samples in GSE17674. (**C**) Functional annotations for 16 ferroptosis regulators. ^*^p < 0.05, ^**^p < 0.01, ^***^p < 0.001, ^****^p = 0. Abbreviation: ns: no significance.

**Figure 4:**
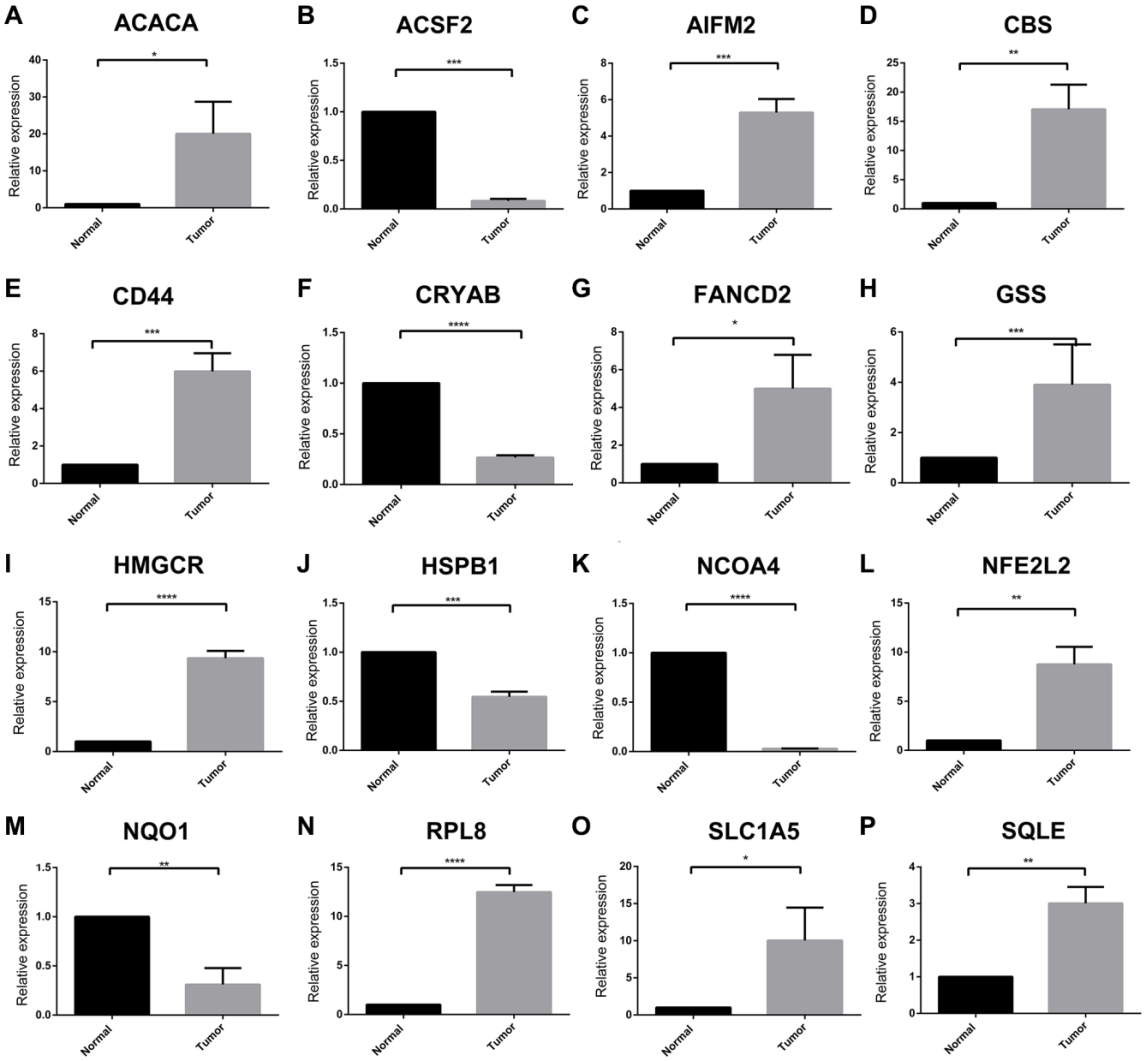
**Expression of each ferroptosis regulators between STS and adjacent tissue by RT-qPCR.** (**A**–**P**) The relative expression of ACACA, ACSF2, AIFM2, CBS, CD44, CRYAB, FANCD2, GSS, HMGCR, HSPB1, NCOA4, NFE2L2, NQO1, RPL8, SLC1A5 and SQLE between tumor and normal tissues, respectively. ^*^p < 0.05, ^**^p < 0.01, ^***^p < 0.001, ^****^p = 0. Abbreviation: ns: no significance.

A total of 60 ferroptosis regulators were screened according to a recent research [23]. After univariate Cox analysis of these 60 regulators in STS, a total of 16 regulators were significantly related to the prognosis of STS and were used for subsequent analysis. The result of univariate Cox regression analysis for prognostic ferroptosis regulators was shown in Figure 2A. A total of 16 ferroptosis regulators were considered to be closely related to the prognosis of STS: SQLE (*p* < 0.001), NFE2L2 (*p* < 0.001), GSS (*p* < 0.001), HSPB1 (*p* = 0.0013), NCOA4 (*p* = 0.002), HMGCR (*p* = 0.0027), CBS (*p* = 0.0063), AIFM2 (*p* = 0.0065), CRYAB (*p* = 0.021), NQO1 (*p* = 0.021), FANCD2 (*p* = 0.024), RPL8 (*p* = 0.027), ACSF2 (*p* = 0.029), ACACA (*p* = 0.031), CD44 (*p* = 0.031), SLC1A5 (*p* = 0.033). Then we explored the somatic mutation of these prognostic ferroptosis regulators and the results were shown in Figure 2B–2D. The main variant classification and type of somatic mutation in STS were missense mutation and single nucleotide polymorphism, respectively. Besides, 6 STS samples had ferroptosis-related mutations, accounting for 2.58% of the total STS samples (*n* = 237). The result of CNV analysis for these ferroptosis regulators was also shown in Figure 2E. CNV widely existed in these ferroptosis regulators. Among them, AIFM2 (56%), NCOA4 (48%) and CRYAB (47%) showed a higher frequency of CNV gain while GSS (40%), SQLE (32%) and RPL8 (30%) exhibited more CNV loss. We further analyzed the relationship between gene expression and CNV and the results were shown in Supplementary Figure 1. CNV was positively correlated with the expression of ferroptosis regulators. The relative location of 16 ferroptosis regulators was also marked in Supplementary Figure 2 and the correlation among these ferroptosis regulators was shown in Figure 3A. Many ferroptosis regulators had significant linear correlations with other ferroptosis regulators at gene expression level. Since huge differences of these ferroptosis regulators were detected in STS through somatic mutation and CNV analysis, we further explored their expression differences between STS and normal skeletal tissue based on public database GSE17674 (Figure 3B). The results of PCA and sample correlation heatmap also showed that ferroptosis could well differentiate the normal and tumor samples (Supplementary Figure 3). The result turned out that most ferroptosis regulators showed significant expression differences. GO functional enrichment analysis was also performed and the result was shown in Figure 3C. The ferroptosis regulators were enriched in response to oxidative stress (*p* < 0.001) and aging (*p* < 0.001). To further explore the trend of these ferroptosis regulators and their potential meaning, we also performed the functional enrichment analysis of ferroptosis regulators that were significantly increased in tumor and normal tissues, respectively. The results showed that ferroptosis regulators up-regulated in tumor tissues were mainly enriched in cholesterol biosynthetic process while ferroptosis regulators up-regulated in normal tissues were mainly enriched in response to oxidative stress (Supplementary Figure 4 and Supplementary Table 5). Moreover, the RT-qPCR was also performed to explore the expression difference of ferroptosis regulators at tissue and cell line levels and the result was shown in Figure 4 and Supplementary Figure 5. Similarly, compared with normal adjacent tissue, ferroptosis regulators exhibited significant expression differences in STS. The above results indicated that ferroptosis had a wide range of differential expression and variation in STS and different ferroptosis modification might regulate the clinical outcome and progression of STS.

### Identification of 2 ferroptosis modification patterns through consensus clustering analysis

**Figure 5:**
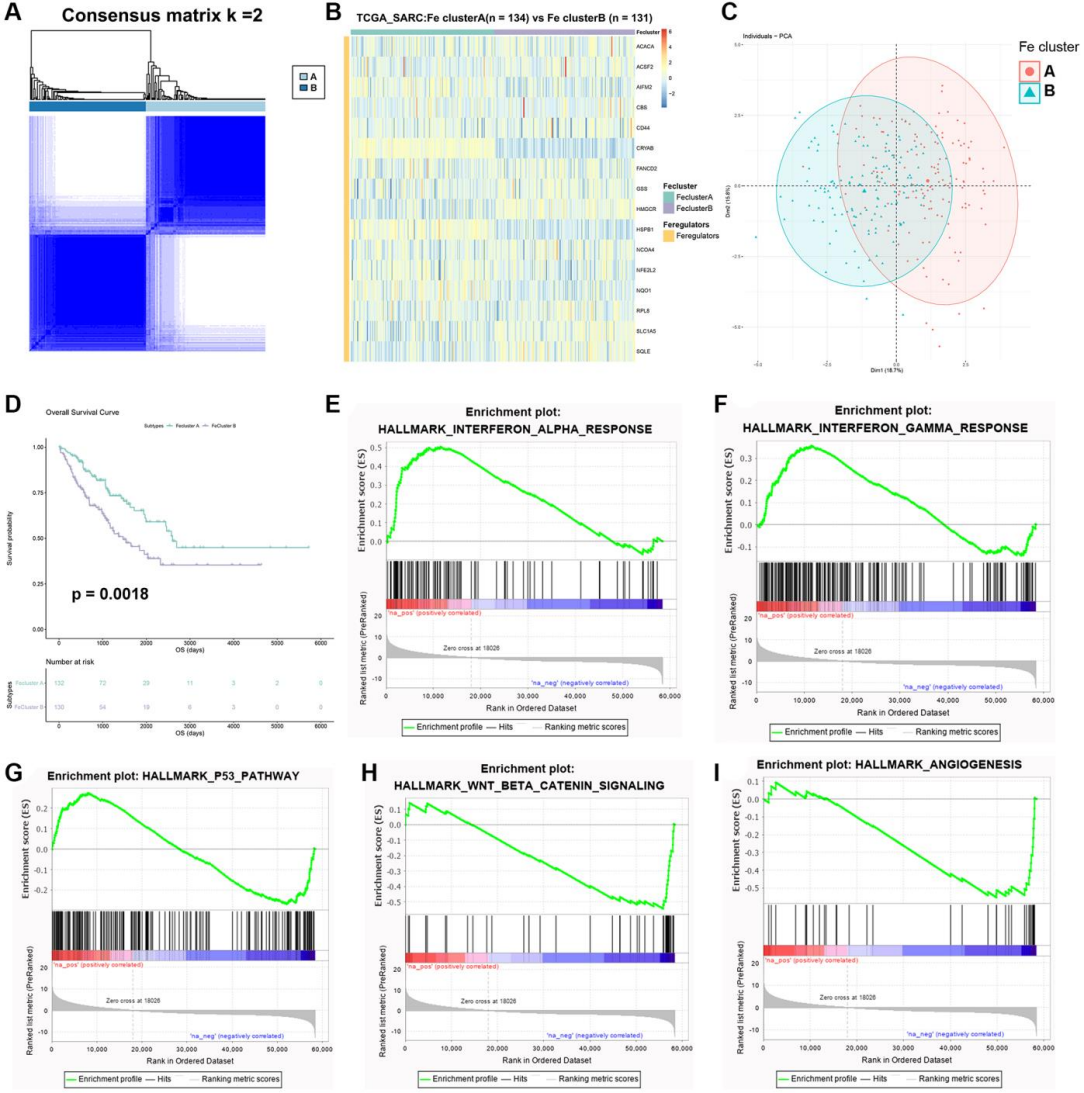
**Identification of two ferroptosis modification patterns in STS.** (**A**) The result of consensus clustering analysis in STS. (**B**) Heatmap of expression of 16 ferroptosis regulators in Fe clusters A and B. (**C**) The result of PCA of two Fe clusters. (**D**) Survival plot of two clusters in TCGA-SARC (*p* = 0.0018). (**E**) Enriched pathways in Fe cluster A: α interferon response. (**F**) Enriched pathways in Fe cluster A: γ interferon response. (**G**) Enriched pathways in Fe cluster A: p53 pathway. (**H**) Enriched pathways in Fe cluster B: WNT signaling. (**I**) Enriched pathways in Fe cluster B: angiogenesis.

To further explore the role of ferroptosis in STS, 2 ferroptosis modification patterns (Fe cluster A and B) were identified through consensus clustering analysis based on the expression of these 16 ferroptosis regulators (Figure 5A). Fe cluster A had 134 samples while Fe cluster B had 131 samples. The expression of 16 ferroptosis regulators between different ferroptosis modification patterns was displayed as heatmap in Figure 5B. AIFM2 (*p* = 0.004), CRYAB (*p* < 0.001), HMGCR (*p* = 0.003), HSPB1 (*p* < 0.001), NFE2L2 (*p* = 0.001), NQO1 (*p* = 0.003), SLC1A5 (*p* < 0.001) and SQLE (*p* < 0.001) were considered significantly differentially expressed between these 2 clusters. PCA also suggested a good clustering effect (Figure 5C). Then survival analysis was performed between Fe cluster A and B and the result was shown in Figure 5D. Compared with Fe cluster B, Fe cluster A showed a significantly better prognosis of STS (*p* = 0.0018). Considering the huge prognostic differences between these 2 clusters, GSEA was further used to explore potential pathways between different ferroptosis modification patterns and the results were shown in Figure 5E–5I. All enriched pathways were shown in Supplementary Tables 6, 7. Fe cluster A was correlated with α interferon (Enrichment score (ES) = 0.50, FDR = 0), γ interferon (ES = 0.36, FDR = 0.001) and p53 pathway (ES = 0.27, FDR = 0.034) while WNT signaling (ES = −0.54, FDR = 0) and angiogenesis (ES = −0.55, FDR = 0) were related to Fe cluster B. These results were consistent with the result of survival analysis, which also proved that different ferroptosis modification patterns affected the prognosis of STS significantly.

### Exploration of immune infiltration between different ferroptosis modification patterns in STS

**Figure 6:**
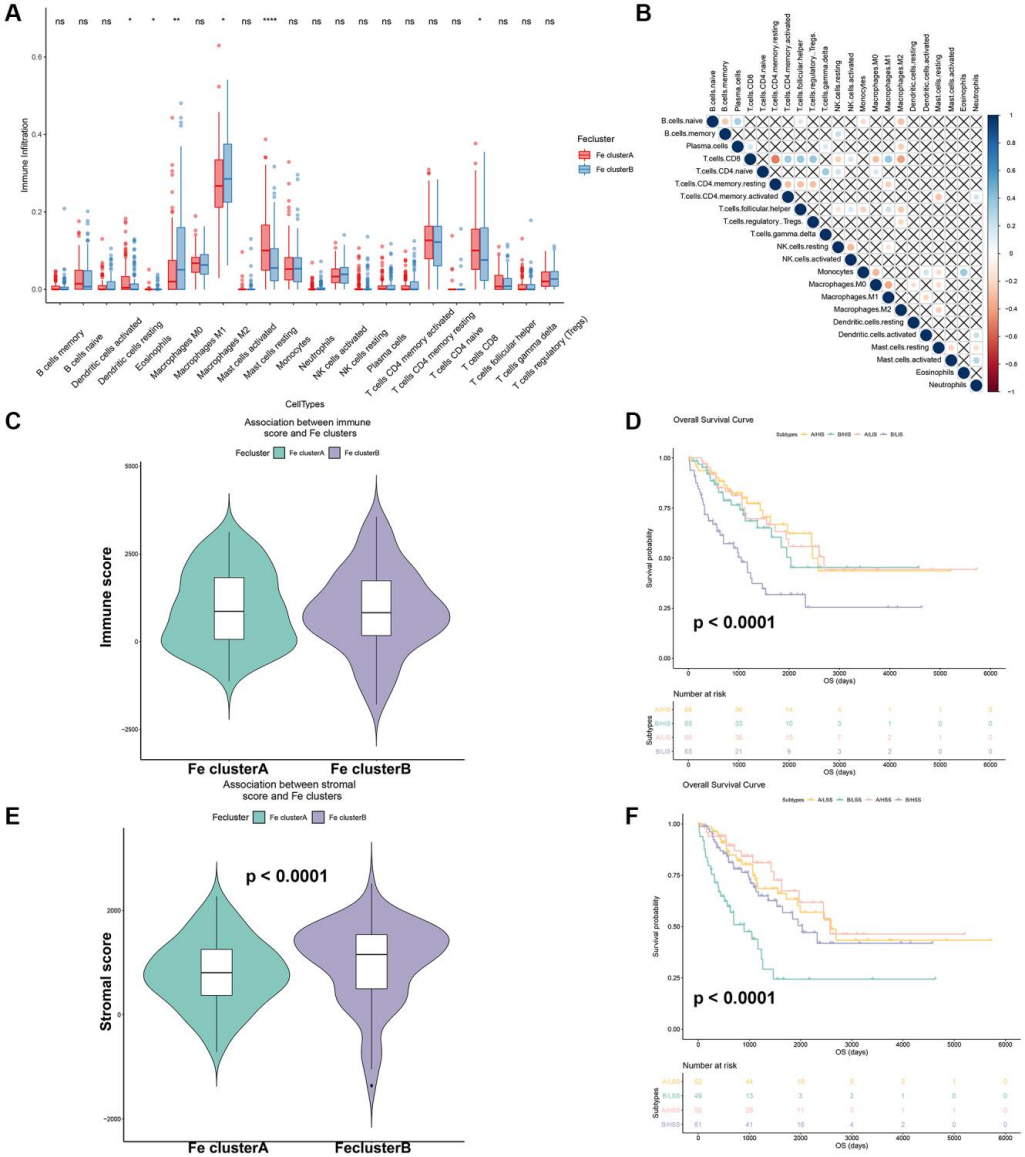
**Effects of different ferroptosis modification patterns on immune infiltration of STS.** (**A**) Comparison of immune cells-infiltration among Fe clusters A and B. (**B**) Correlation plot of each immune cells in TCGA-SARC. (**C**) Comparison of immune score among Fe clusters A and B. (**D**) Survival analysis of different immune scores among Fe clusters A and B (A: Fe cluster A, B: Fe cluster B, Abbreviations: LSS: Low immune score; HSS: High immune score); (**E**) Comparison of stromal scores among Fe clusters A and B. (**F**) Survival analysis of different stromal scores among Fe clusters A and B. (A: Fe cluster A, B: Fe cluster B, Abbreviations: LIS: Low stromal score; HIS: High stromal score); ^*^p < 0.05, ^**^p < 0.01.

In order to explore the role of ferroptosis in TME infiltration, a key factor regulating tumor immunotherapy, general immune infiltration of STS samples from TCGA-SARC were displayed in Supplementary Figure 6. Moreover, the 22 immune cells infiltration of different ferroptosis modification were shown in Figure 6A. Among them, dendritic resting cells (*p* < 0.05), eosinophils (*p* < 0.05), CD8 T cells (*p* < 0.05) were found to have a higher expression in Fe cluster A. Meanwhile, mast resting cells (*p* < 0.0001), M0 (*p* < 0.01) and M2 macrophages (*p* < 0.05) were highly expressed in Fe cluster B. The correlation plot of these 22 immune cells were also displayed in Figure 6B. Besides, the immune score and stromal score of different ferroptosis modification were calculated. In Figure 6C, there was no significant difference in immune scores between the two ferroptosis modification patterns. In Figure 6D, the stromal score between two ferroptosis modification patterns showed statistically significant (*p* = 0.004). Moreover, the survival curve for different immune/stromal score in different ferroptosis modification patterns were displayed in Figure 6E, 6F. Different immune score/ stromal score showed significant prognostic differences in different ferroptosis modification patterns (*p* < 0.0001). The above results revealed that different ferroptosis modification patterns affected the STS microenvironment and had a significant impact on the prognosis of STS.

### Establishment of the Fescore

**Figure 7:**
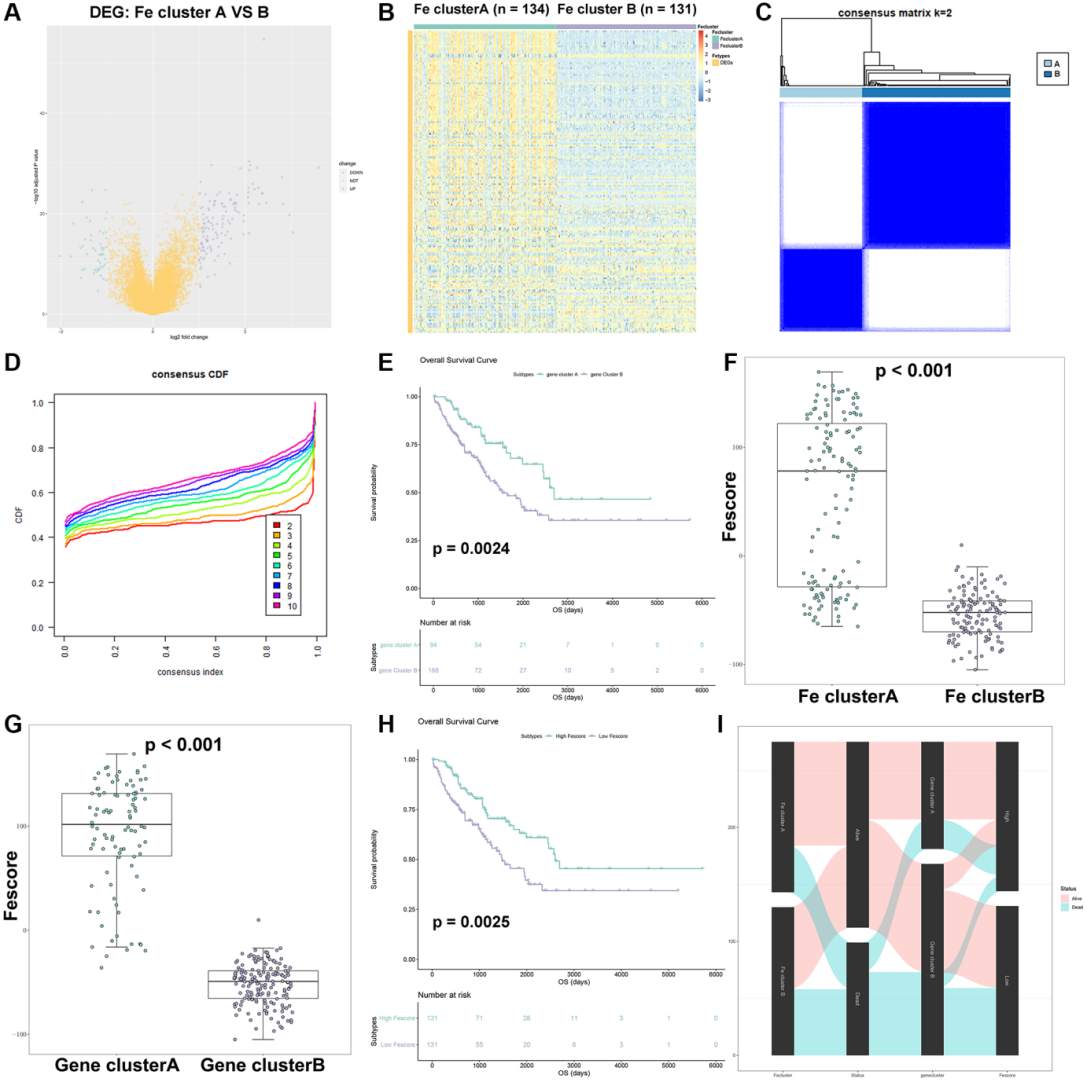
**Construction of Fescore.** (**A**) Volcano plot of DEGs between Fe cluster A and B. (**B**) The heatmap of expression of DEGs in Fe cluster A and B. (**C**) The result of consensus clustering analysis in STS based on 143 DEGs; The relationship between DEGs and these prognostic genes visualized as a Sankey diagram. (**D**) The cumulative distribution function plot in TCGA-SARC based on 143 DEGs. (**E**) Survival plot of gene clusters A and B in TCGA-SARC (*p* = 0.0024). (**F**) Comparison of Fescore among Fe clusters A and B. (**G**) Comparison of Fescore among gene clusters A and B. (**H**) Survival plot of high and low Fescore in TCGA-SARC (*p* = 0.0025). (**I**) The relationship between Fe clusters, gene clusters survival status and Fescore visualized as a Sankey diagram.

In order to further verify the stability of consensus clustering in STS, DEG analysis was performed between two Fe clusters and the result was shown in Figure 7A. 125 genes were upregulated in Fe cluster A while 49 genes were upregulated in Fe cluster B. The full list of these DEGs were shown in Supplementary Table 8. Functional enrichment analysis was performed based on these DEGs and the results showed that these DEGs were enriched in oxidoreductase activity-related pathways, which was necessary for ferroptosis occurrence (Supplementary Figure 7 and Supplementary Table 9). The heatmap of these DEGs was also displayed in Figure 7B. These DEGs were further used for cMap analysis and the results were shown in Supplementary Table 10. Among them, sulfadimethoxine (*p* < 0.001) and withaferin A (*p* = 0.016) might be potential targets treating STS. Then, consensus clustering analysis was performed based on the expression of these 174 DEGs and the results were shown in Figure 7C, 7D. Similarly, STS samples were divided into 2 new clusters (gene cluster A and B). Gene cluster A had 95 samples while gene cluster B had 170 samples. After comparing the results of two consensus clustering, 96.8% of samples from gene cluster A were found in Fe cluster A while 75.3% of samples from gene cluster B were also found in Fe cluster B. These results indicated that our clustering result was relatively reliable. Further, the survival analysis between gene cluster A and B was performed (Figure 7E). Gene cluster A showed a better clinical outcome than gene cluster B (*p* = 0.0024). Besides, 174 DEGs were used for univariate Cox regression analysis to screen prognostic DEGs. A total of 43 prognostic DEGs were identified and the result was shown in Supplementary Table 11. Then the Fescore of each STS sample was calculated through PCA and the results were shown in Figure 7F, 7G. Fe cluster A exhibited a significantly higher Fescore than Fe cluster B (*p* < 0.001). Similarly, gene cluster A also had a significantly higher Fescore than gene cluster B (*p* < 0.001). After STS samples were divided into high and low Fescore groups, the corresponding survival analysis results were shown in Figure 7H. High Fescore group showed a significantly better prognosis of STS than low Fescore (*p* = 0.0025). Finally, a Sankey chart (Figure 7I) was drawn to summarize our above findings: Fe cluster A and gene cluster A were related to a better prognosis of STS and high Fescore while Fe cluster B and gene cluster B were related to a poorer outcome of STS and lower Fescore. The Fescore could quantify ferroptosis modification in STS and well indicate the clinical outcome of STS.

### Fescore was related to the prognosis of multiple tumors

**Figure 8:**
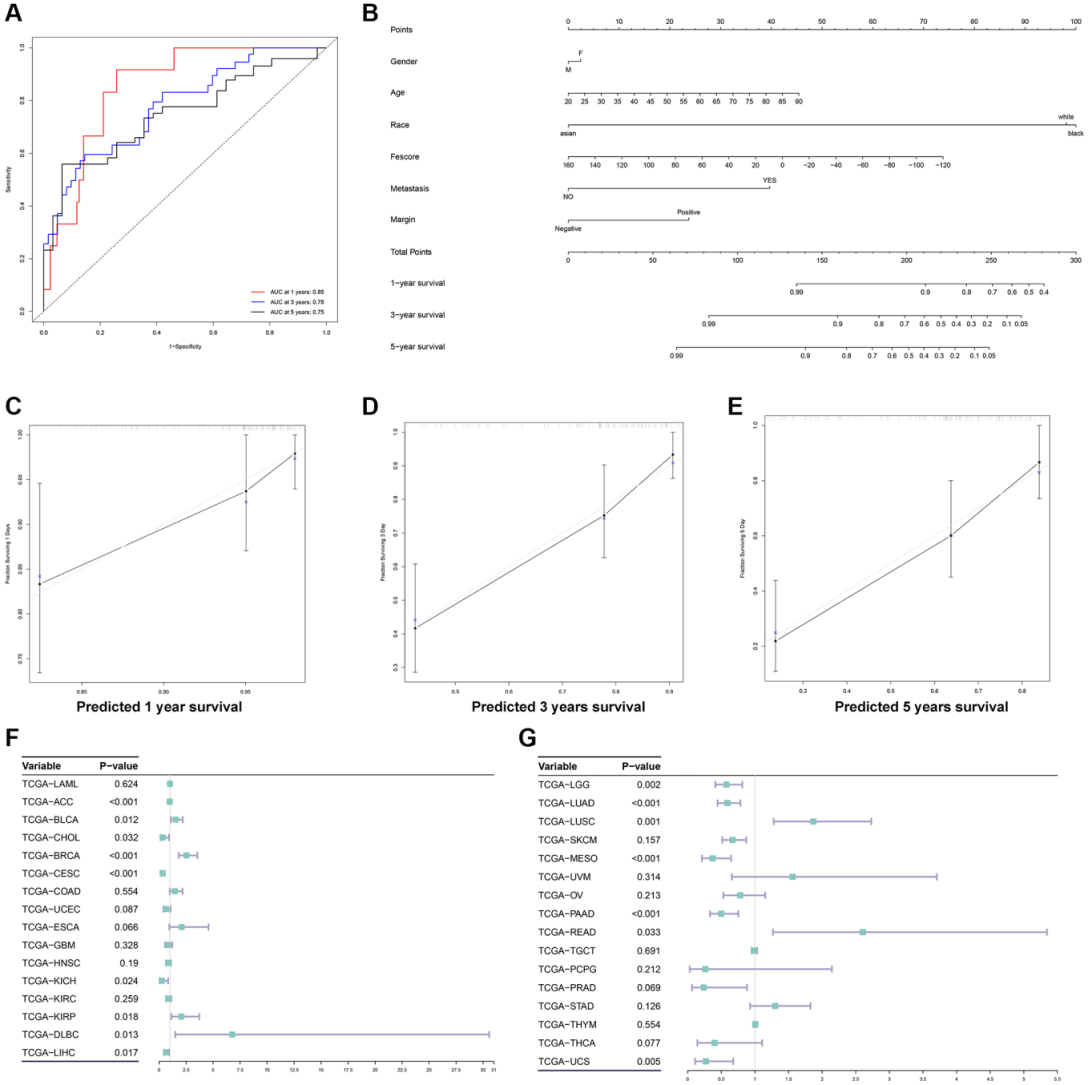
**Construction of a prognostic nomogram and validation of the Fescore in multiple tumors.** (**A**) ROC curve of Fescore for predicting the 1-year, 3- and 5-years survival of STS. (**B**) The prognostic nomogram based on gender, age, race, metastasis, margin status and Fescore for predicting the prognosis of STS. (**C**–**E**) The calibration curve of 1-year, 3- and 5-years survival of STS. (**F**, **G**) Validation of Fescore across 33 types of tumors.

To further evaluate the prognostic value of Fescore in STS, the ROC curve of Fescore for predicting the survival of STS was shown in Figure 8A. Area under curve of Fescore predicting the 1 year, 3- and 5-years survival of STS were 0.85, 0.78, 0.75, respectively. Besides, a prognostic nomogram based on gender, age, race, metastasis, margin status and Fescore was also constructed (Figure 8B). The corresponding calibration curve of 1 year, 3- and 5-years survival of STS were also displayed in Figure 8C–8E. The nomogram could effectively predict the prognosis of STS. Besides, we further explored the prognostic value of Fescore in other 32 tumors from TCGA database. The results of univariate Cox analysis of Fescore in different tumors were shown in Figure 8F, 8G. Among them, Fescore was significantly related to the prognosis of TCGA-ACC (*p* < 0.001), TCGA-BLCA (*p* = 0.012), TCGA-CHOL (*p* = 0.032), TCGA-BRCA (*p* < 0.001), TCGA-CESC (*p* < 0.001), TCGA-KICH (*p* = 0.024), TCGA-KIRP (*p* = 0.018), TCGA-DLBC (*p* = 0.013), TCGA-LIHC (*p* = 0.017), TCGA-LGG (*p* = 0.002), TCGA-LUAD (*p* < 0.001), TCGA-LUSC (*p* = 0.001), TCGA-MESO (*p* < 0.001), TCGA-PAAD (*p* < 0.001), TCGA-READ (*p* = 0.033) and TCGA-UCS (*p* = 0.005). We also tested the Fescore on different subtypes of STS including undifferentiated sarcoma, Liposarcoma, Leiomyosarcoma, Synovial Sarcoma, Ewing Sarcoma, and Fibrosis sarcoma through survival analysis, tumor grading, CINSARC index and metastasis analysis and results were shown in Supplementary Figure 8. While the significant differences between high and low Fescore groups were not detected in liposarcoma, Fescore could be applied to the majority of STS subtypes and well predicted its prognosis and clinical characteristics. The above results showed that the Fescore could serve as a prognostic predictor in a variety of tumors.

### Fescore effectively predicted immunotherapy response

**Figure 9:**
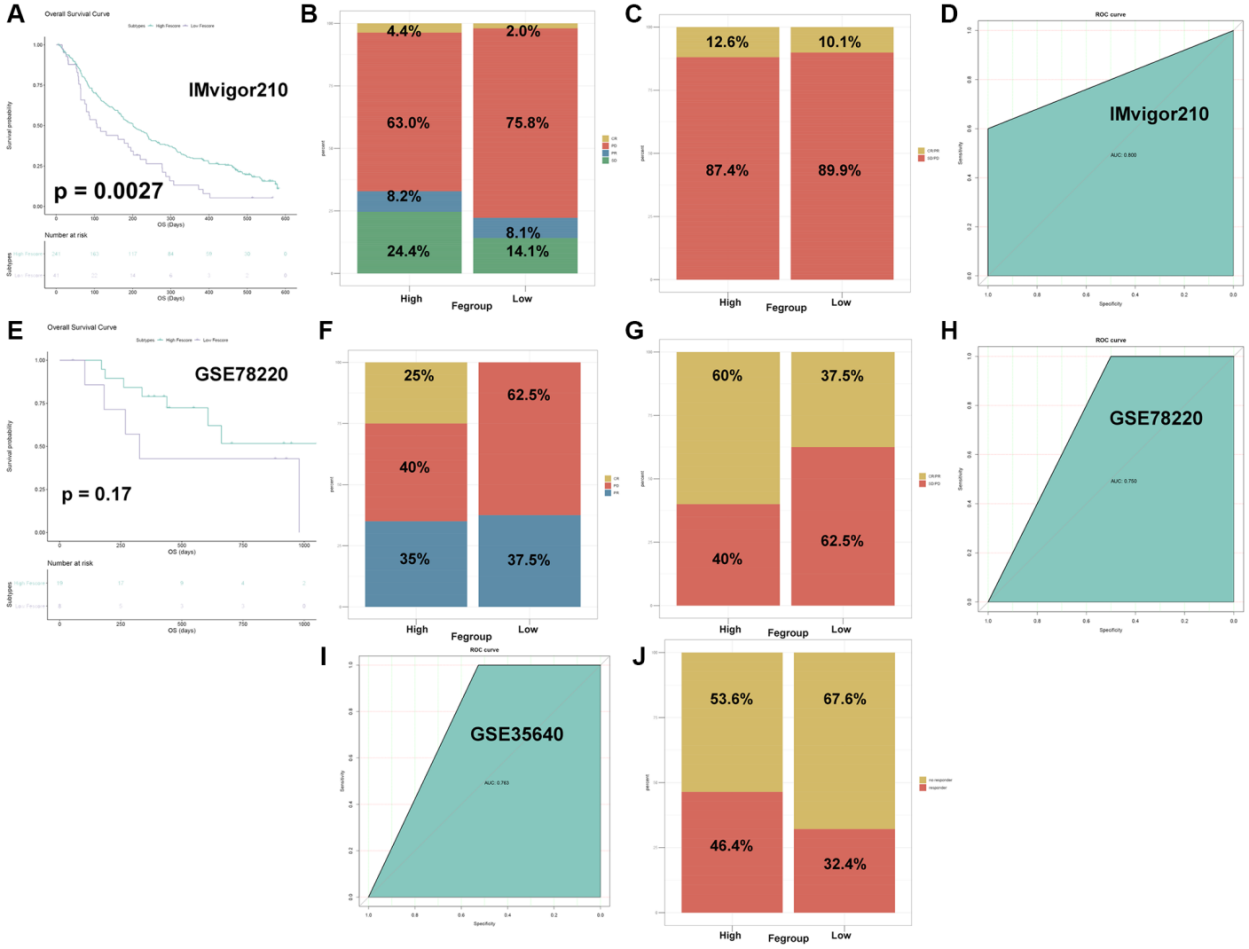
**The Fescore predicted immune response in immunotherapy datasets.** (**A**) Survival plot of high and low Fescore in IMvigor210 dataset (*p* = 0.0027). (**B**) Relative percent of immune responses in high and low Fescore in IMvigor210 dataset. Abbreviations: CR: complete response; PR: partial response; SD: stable disease; PD: progressive disease. (**C**) Relative percent of CR/PR and SD/PD in high and low Fescore in IMvigor210 dataset. (**D**) ROC curve for prediction of immune response in IMvigor210 dataset. (**E**) Survival plot of high and low Fescore in GSE78220 dataset (*p* = 0.17). (**F**) Relative percent of immune responses in high and low Fescore in GSE78220 dataset. Abbreviations: CR: complete response; PR: partial response; PD: progressive disease. (**G**) Relative percent of CR/PR and SD/PD in high and low Fescore in GSE78220 dataset. (**H**) ROC curve for prediction of immune response in GSE78220 dataset. (**I**) ROC curve for prediction of immune response in GSE35640 dataset. (**J**) Relative percent of CR/PR and SD/PD in high and low Fescore in GSE35640 dataset.

Next, we explored the role of Fescore in predicting tumor immunotherapy response. The Fescore was calculated for each sample in immunotherapy dataset IMvigor210 (PD-L1). Then survival curve among high and low Fescore groups was shown in Figure 9A. High Fescore group showed a better prognosis than low Fescore group (*p* = 0.0027). The relative percent of complete response (CR), Progressive disease (PD), partial response (PR) and stable disease (SD) in high Fescore group were 4.4%, 63.0%, 8.2% and 24.4%, respectively. Meanwhile in low Fescore group the relative percent of CR, PD, PR and SD were 2.0%, 75.8%, 8.1% and 14.1%, respectively. The relative percent of CR/PR in high Fescore group and low Fescore group were 12.6% and 10.1%, respectively (Figure 9B, 9C). These results indicated that high Fescore was correlated with a better immunotherapy outcome. The AUC of Fescore for predicting the immunotherapy response in IMvigor210 was 0.800 (Figure 9D).

Besides, the Fescore of each sample in immunotherapy dataset GSE78220 (PD-1) was also calculated. Compared with high Fescore group, low Fescore group exhibited a poorer prognosis (Figure 9E, *p* = 0.17). The relative percent of CR, PD, PR in high Fescore group were 25%, 40% and 35%, respectively. The proportion of PD and PR in low Fescore group were 62.5% and 37.5%, respectively. In high Fescore group CR/PR accounted for 60% while in low Fescore group CR/PR accounted for 37.5% (Figure 9F, 9G). The AUC of Fescore for predicting the immunotherapy response in GSE78220 was 0.750 (Figure 9H).

Moreover, the Fescore of samples from immunotherapy dataset GSE35640 (MAGE-A3) was calculated. In high Fescore group, 46.4% of the patients responded to immunotherapy while in low Fescore group this number dropped to 32.1% (Figure 9I). The AUC of Fescore for predicting the immunotherapy response in GSE35640 was 0.763 (Figure 9J). The above results show that Fescore had a good predictive effect in different immunotherapy response.

### The downregulation of LOX inhibited the biological behavior of STS cells and increased the production of ROS

**Figure 10:**
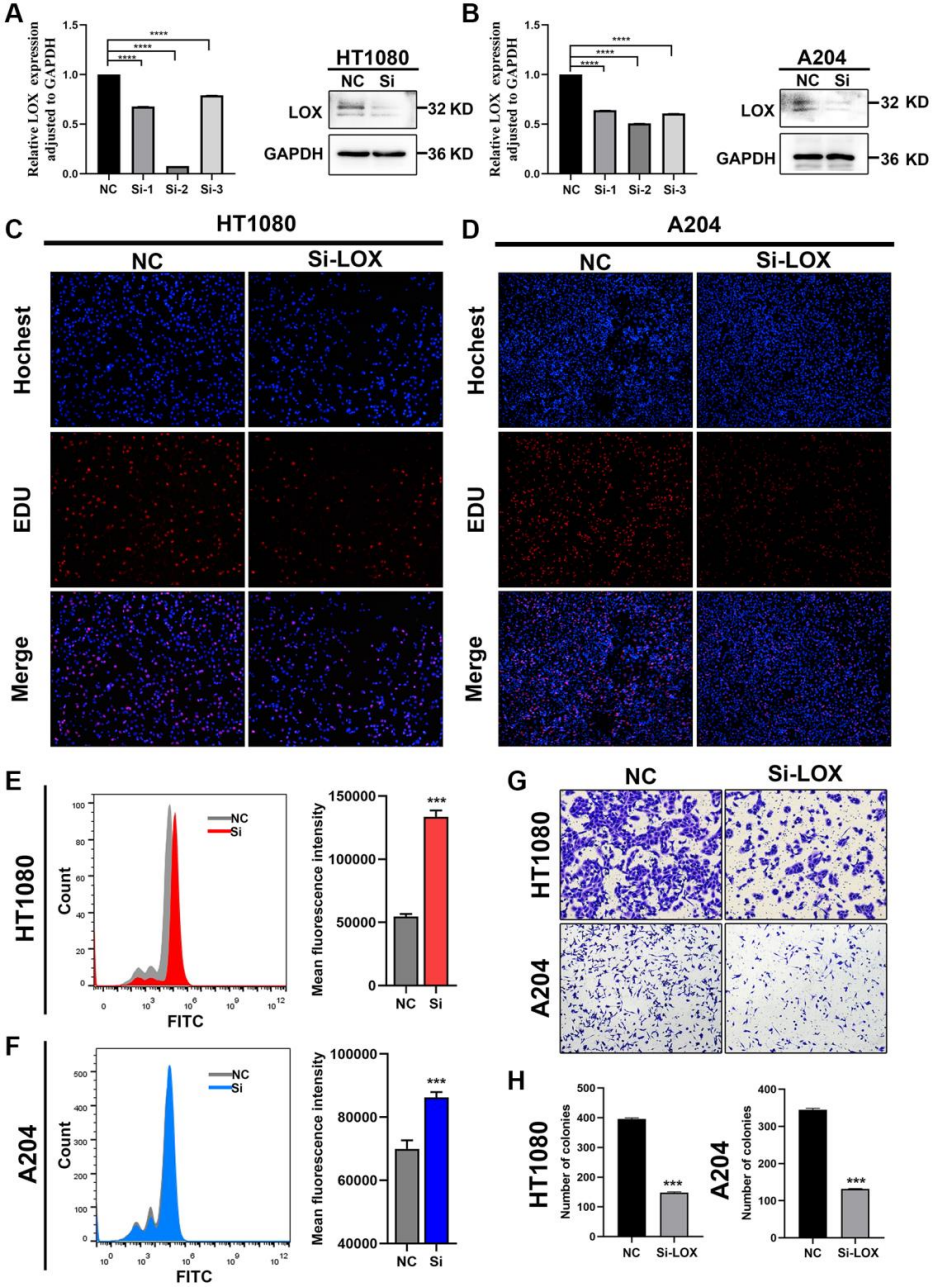
**Downregulation of LOX inhibits growth and increases ROS production in HT1080 and A204 cells.** (**A**, **B**) qPCR analysis and Western blotting analysis were performed to detect the mRNA and protein levels of LOX in HT-1080 and A204 cells. (**C**, **D**) EdU assay was used to detect the proliferation of HT-1080 and A204 cells. (**E**, **F**) ROS levels were analyzed by flow cytometry in HT-1080 and A204 cells. (**G**, **H**) Transwell assay was performed to assess migration of HT-1080 and A204 cells. ^***^p < 0.001, ^****^p = 0.

In order to further prove the robustness of Fescore, we performed experiments to verify the function of hub gene that constructed the Fescore in STS and its relationship with ferroptosis. First, we conducted protein-protein interaction analysis among the genes that constructed Fescore and further screened LOX as a hub gene based on degree more than 4 (Supplementary Figure 9). Then, we focused on whether LOX played an important role in ferroptosis. Then, the expression of LOX was knockdown in HT-1080 and A204 cells (Figure 10A, 10B). And EdU assay were used to evaluate the proliferative capacity of HT-1080 and A204 cells. When LOX was knocked down, the proliferation of HT-1080 and A204 cells were decreased (Figure 10C, 10D). Besides, ROS levels in HT-1080 and A204 cells were increased after LOX knockdown (Figure 10E, 10F). Furthermore, the HT-1080 and A204 cells migration were assessed by Transwell assay and the results showed that knockdown of LOX were inhibited the migration of HT-1080 and A2044 cells (Figure 10G, 10H).

## DISCUSSION

Although STS is a rare tumor originated from mesenchymal tissue, there has been few breakthroughs to improve the prognosis of STS in recent decades. Ferroptosis, a special way of cell death, has been gradually found in a variety of cancer cells and promoting ferroptosis in tumor cells has become a novel treatment for cancer. For example, decreased KLF2 attenuated ferroptosis in renal cell carcinoma by regulating GPX4 level [40]. Induced ferroptosis in gastric cancer could also lead to the higher sensitivity of gastric cancer cells to cisplatin [41]. However, the role and modification characteristics of ferroptosis in STS are still unclear. Therefore, it is urgent to clarify the classification of STS related to ferroptosis and quantify the modification of ferroptosis in STS.

In this study, we screened 16 ferroptosis regulators that were significantly related to the prognosis of STS, and further found that they showed great differences in both expression level and variation level. The functional enrichment analysis indicated differentially expressed ferroptosis regulators were enriched in cholesterol and glutathione biosynthetic processes and response to oxidative stress. A recent study revealed that glutathione biosynthesis and oxidative stress were closely related to the progression of ferroptosis [42]. In addition, previous research also reported that high cholesterol could make tumor cells resistant to ferroptosis, thus promoting tumor development [43]. Therefore, the differentially expressed ferroptosis regulators between normal and tumor tissues might also regulate ferroptosis through these pathways. SQLE, NFE2L2 and GSS were the ferroptosis regulators most related to the prognosis of STS. Further, the result of CNV implied that GSS and AIFM2 had the highest frequency of variation. A recent study showed that decreased expression of SQLE could lead to limited growth of liver cancer through p53 pathway [44]. Recent research indicated that reduced expression of NFE2L2 was related to a better prognosis of breast cancer [45]. In Chen’s study GSS was also determined as a potential target against tumor by regulating polydatin [46]. Increased GSS was also found to enhance the resistance to cisplatin in ovarian cancer [47]. Activated AIFM2 could also accelerate the apoptosis in lung cancer [48]. These literature reports were consistent with our analysis, which also suggested that these ferroptosis regulators might be important for STS progression.

By using consensus clustering, we identified two different ferroptosis modification patterns in STS. These two Fe clusters showed significant differences in prognosis and enrichment pathways. Fe cluster A was correlated with p53 pathway and had a better prognosis of STS while Fe cluster B was correlated with angiogenesis and MYC pathway and showed a poorer outcome of STS. P53 pathway is a recognized pathway that inhibit tumor progression [49]. Angiogenesis has also been proven to be a key part of tumor progression. A recent study reported that angiogenesis was necessary for bladder cancer progression [50]. MYC is an oncogene accelerating tumor progression. A latest study revealed that MYC could inhibit tumor immunity in neuroblastoma and melanoma [51]. The immune infiltration among different ferroptosis modification revealed that M2 macrophage was enriched in Fe cluster B while CD8 T cell was enriched in Fe cluster A. M2 macrophages have been proved to promote tumor development in many studies. A recent study implied that M2 macrophage polarization induced by FNDC5 was associated with hepatocellular carcinoma cell growth [52]. A former study also reported that M2 macrophage was related to poor prognosis of lung cancer [53]. These reports supported our results and indicated that different ferroptosis modification patterns were correlated with the prognosis and immune infiltration of STS.

The cMap analysis screened sulfadimethoxine and withaferin A based on different ferroptosis modification patterns. A former study reported that sulfadimethoxine could be used as potential drugs against breast cancer and colon cancer [54]. Withaferin A, a promising compound with anti-tumor potential, has been studied in many fields. A recent article showed that withaferin A could be a potential target treating breast cancer [55]. Moreover, withaferin A could also promote apoptosis in osteosarcoma cell [56]. These drugs might be potential targets against STS. The Fe clusters and gene clusters determined based on DEGs had a great intersection with each other, indicating the consensus clustering was relatively stable. Besides, high Fescore group showed a significantly better prognosis of STS than low Fescore. The prognostic nomogram also effectively predicted the survival of STS and the ROC curve of Fescore for predicting the prognosis of STS showed that Fescore was a good prognostic predictor. Fescore was also significant correlated with the prognosis of 16 other tumors from TCGA. Fescore played a protective role in some tumors while in other tumors, Fescore was a risk factor, which might be due to the heterogeneity between different tumors.

Tumor immunotherapy has been controversial because of its limited application and high price. Therefore, exploring a good predictor to predict the outcome and prognosis of tumor immunotherapy is of great significance. Here we reported the Fescore could well predict the immunotherapeutic response in different kinds of immunotherapy (MAGE-A3, PD-1/L1). High Fescore also was correlated with better immunotherapeutic response and prognosis. Hence, the Fescore could also be used to predict the response to tumor immunotherapy, which contribute to the clinical application of tumor immunotherapy in the future.

Our further experiments also showed that LOX, as the hub gene in Fescore, could regulate the STS progression. Iron is involved in the activation of LOX, an iron-containing enzyme that promotes the generation of lipid ROS such as lipid peroxides [57]. Increased lipid peroxidation subsequently leads to damage and rupture of cellular and mitochondrial membranes, causing ferroptosis. EDU proliferation experiments and Transwell experiments confirmed that LOX had an important value in STS. The ROS experiment showed that LOX played a role through ferroptosis and affected the prognosis of tumors, which was also consistent with Shintoku’s reports [57].

Although we determined different modifications of ferroptosis in STS and established the Fescore to quantify the modification of ferroptosis, our study still had limitations. First of all, the modifications of ferroptosis in STS were identified by public datasets, and more clinical samples were needed to perform transcriptome sequencing to validate the Fescore. Secondly, we also noticed that due to different sequencing methods, population baselines (race, gender, stage, etc.), and sampling methods, there was some heterogeneity among datasets. Although the Fescore could well predict the prognosis and immunotherapy response in different cohorts, larger STS cohorts are needed for further research. Thirdly, due to the difference of subtypes of STS, few immune cells had different infiltration between the ferroptosis modification patterns. We are currently collecting clinical STS samples from our department and will continue to conduct ferroptosis-related research. We also hope more researchers will pay attention to this field.

## CONCLUSION

In summary, we identified different modifications of ferroptosis in STS and constructed the Fescore to quantify the modification of ferroptosis. The Fescore was significantly related to the immune infiltration and prognosis of STS and immunotherapy response. The Fescore was also successfully validated in multiple tumors and STS subtypes. Therefore, the Fescore could provide a new prospect for precision medicine and tumor immunotherapy.

## Supplementary Materials

Supplementary Figures

Supplementary Tables 1-7 and 9-11

Supplementary Table 8
