# Supplementary material for: Molecular characterization of ferroptosis in soft tissue sarcoma constructs a prognostic and immunotherapeutic signature through experimental and bioinformatics analyses
Source: Aging (Albany NY). 2023 Oct 20;15(20):11412–47. doi: 10.18632/aging.205133 (PMC10637810; doi:10.18632/aging.205133)
Supplement: Supplementary Figures [file aging-15-205133-s001.pdf]

## SUPPLEMENTARY FIGURES

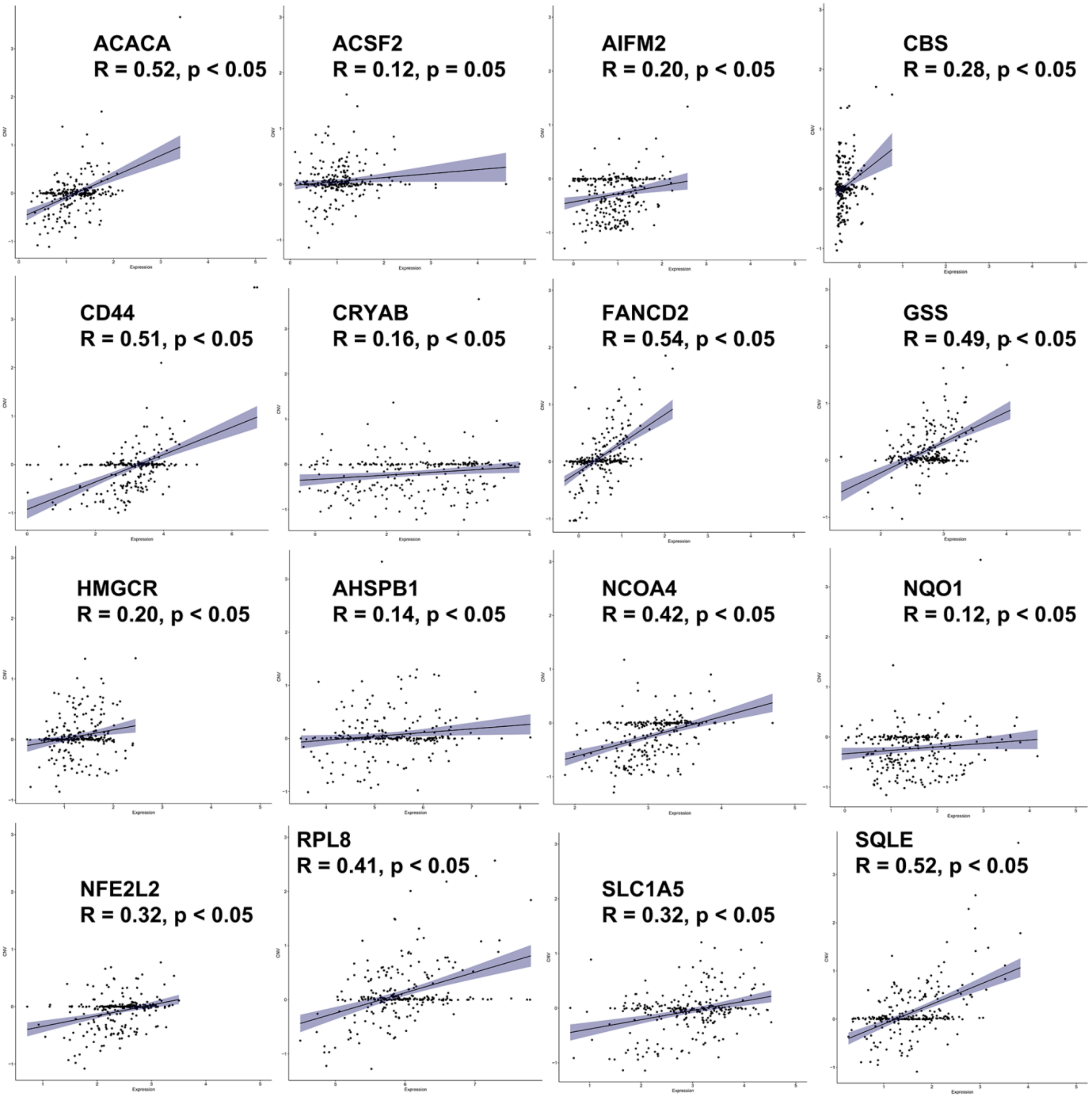


**Supplementary Figure 1. The relationship between CNV and expression of ferroptosis regulators.**

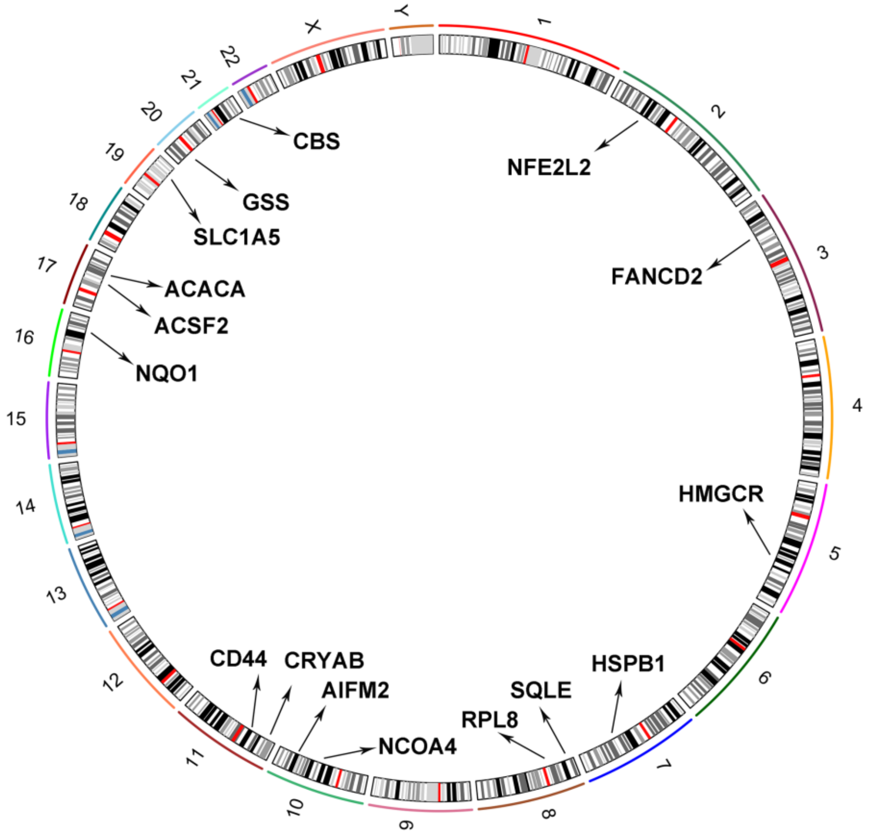


**Supplementary Figure 2. The relative location of 16 ferroptosis regulators.**

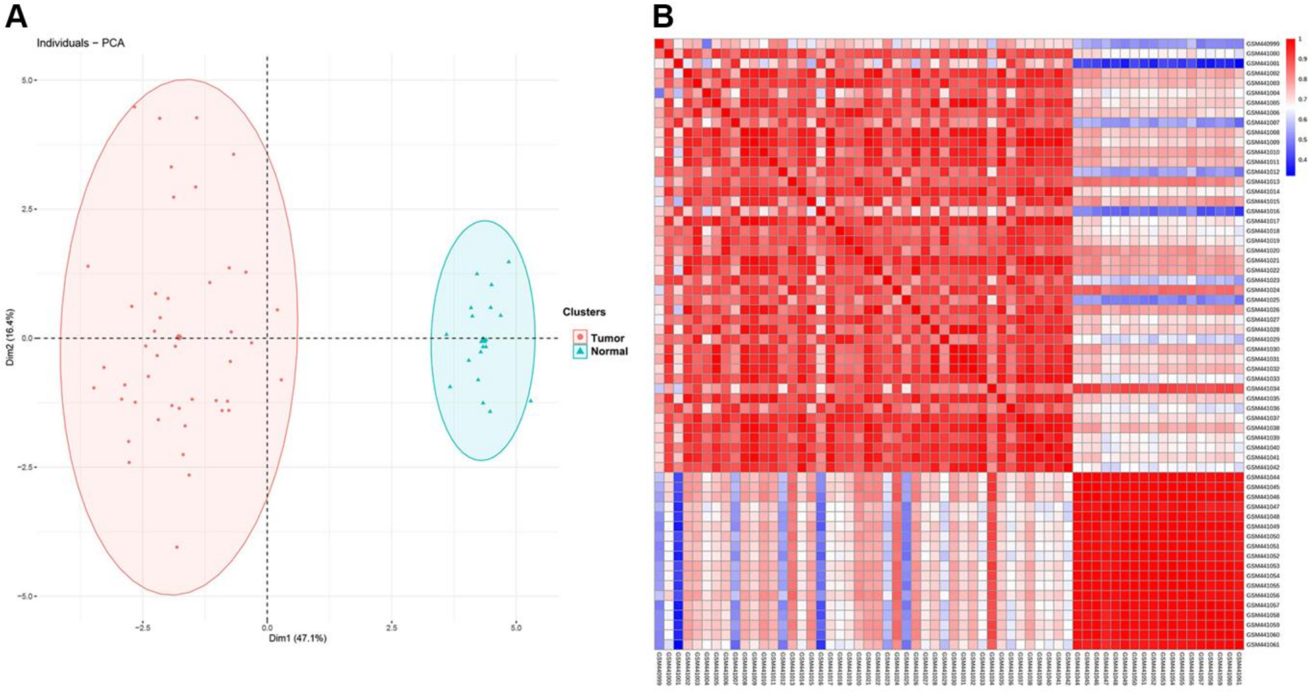


**Supplementary Figure 3.** The PCA (**A**) and sample correlation heatmaps (**B**) of normal and tumor samples based on expression of ferroptosis regulators.

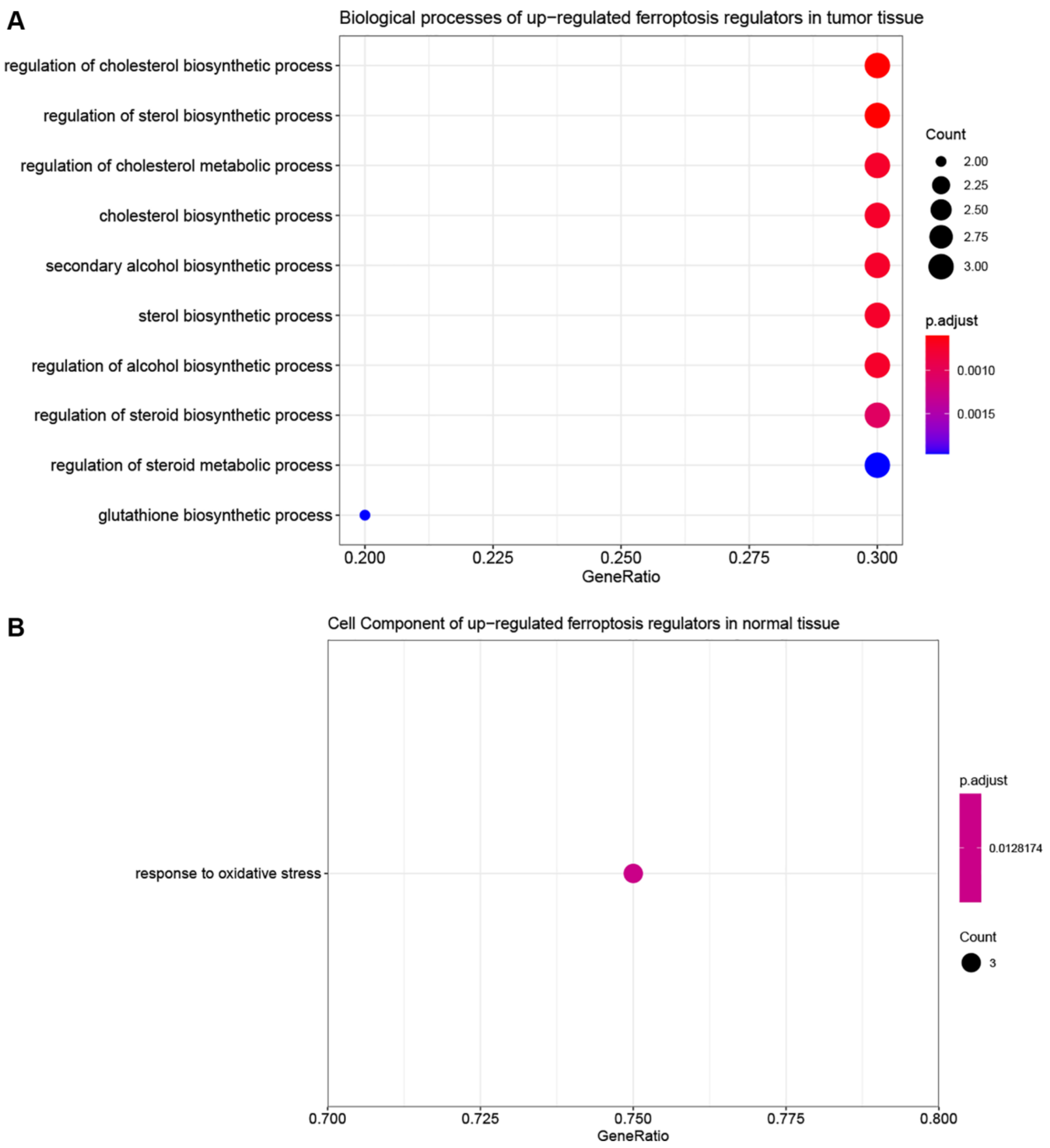


**Supplementary Figure 4. Functional enrichment analysis of differentially expressed ferroptosis regulators between normal and tumor tissues.** (**A**) Biological processes of upregulated ferroptosis regulators in tumor tissue. (**B**) Cell Component of up-regulated ferroptosis regulators in normal tissue.

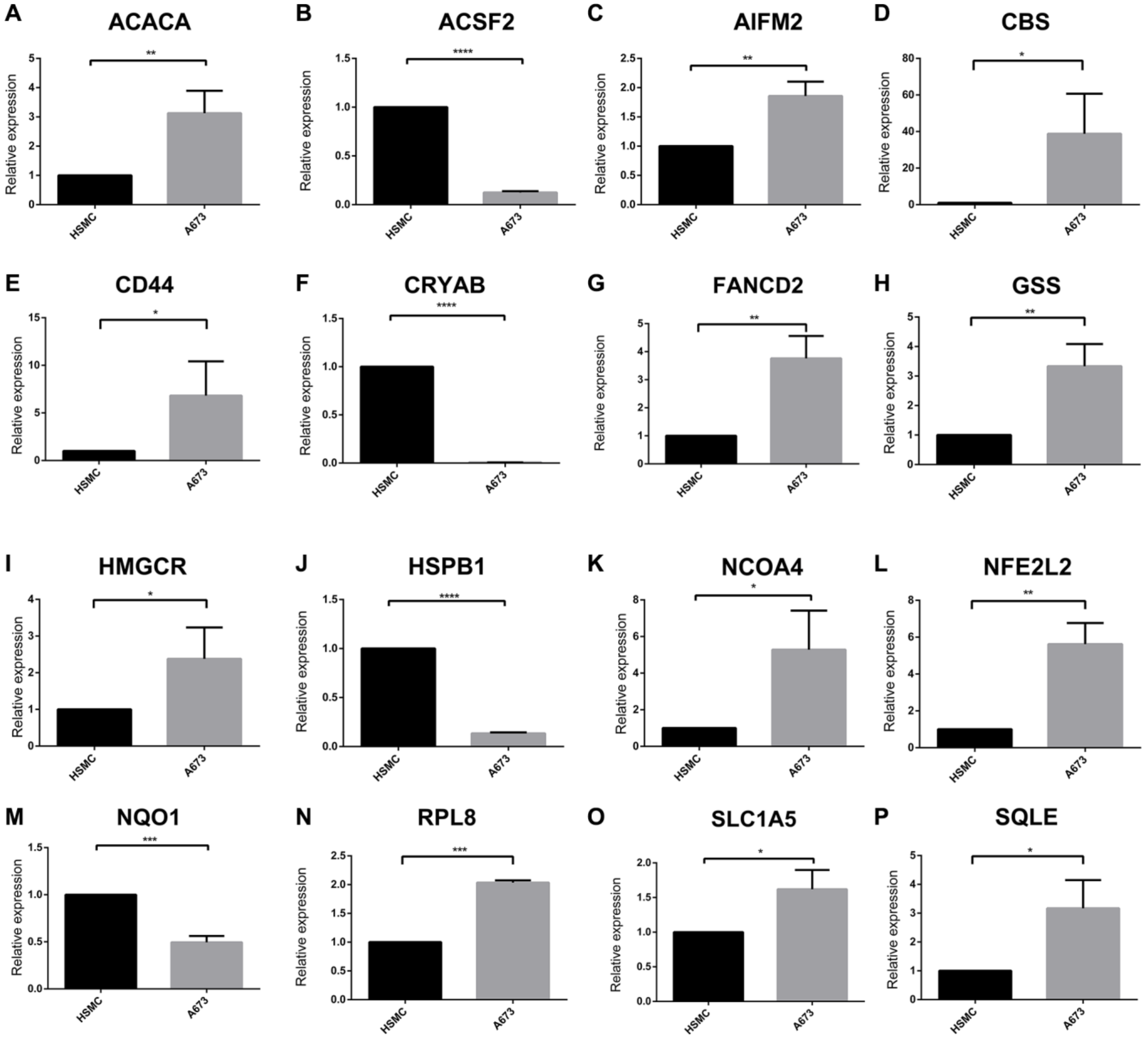


**Supplementary Figure 5. Expression of each ferroptosis regulators between STS cell and normal skeletal cell by RT-qPCR.** (**A**–**P**) The relative expression of ACACA, ACSF2, AIFM2, CBS, CD44, CRYAB, FANCD2, GSS, HMGCR, HSPB1, NCOA4, NFE2L2, NQO1, RPL8, SLC1A5 and SQLE between tumor and normal cells, respectively. $^{*}p < 0.05$, $^{**}p < 0.01$, $^{***}p < 0.001$, $^{****}p = 0$.

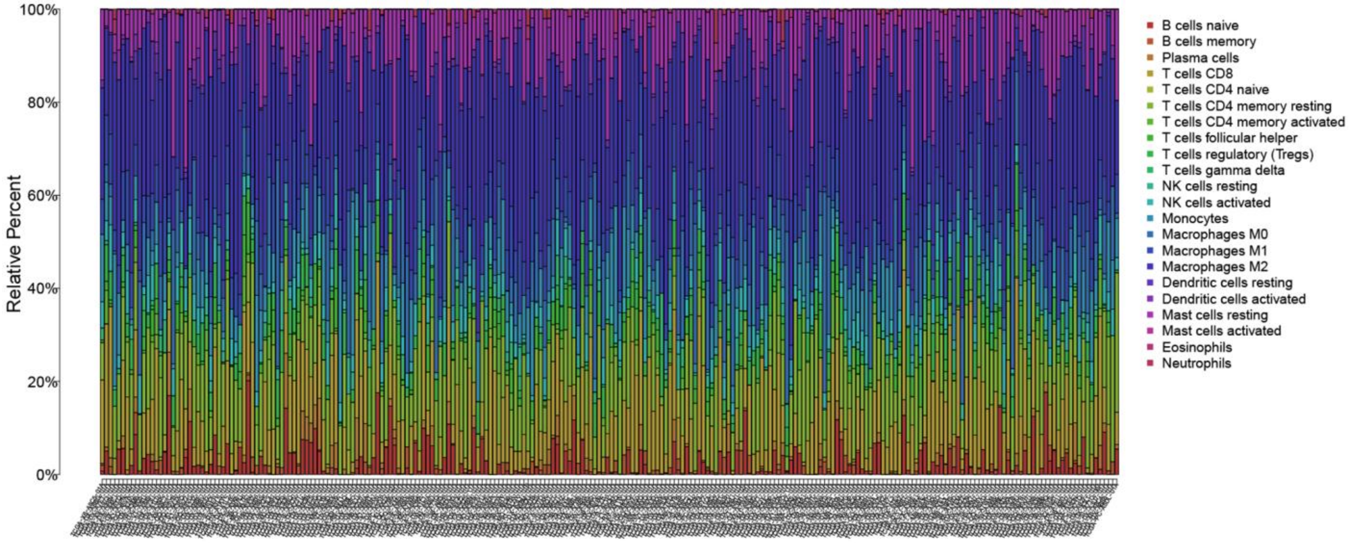


**Supplementary Figure 6. Immune infiltration of each sample in TCGA-SARC.**

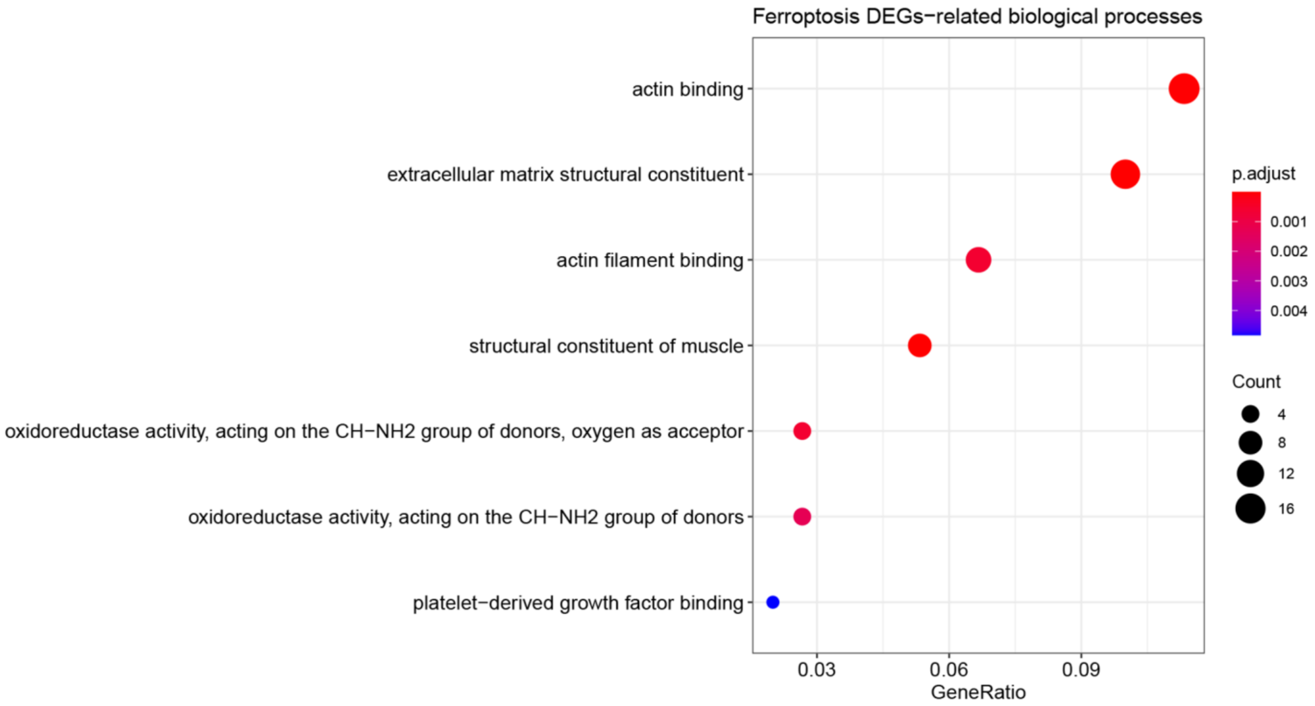


**Supplementary Figure 7. Functional enrichment analysis of DEGs between two ferroptosis modification patterns.**

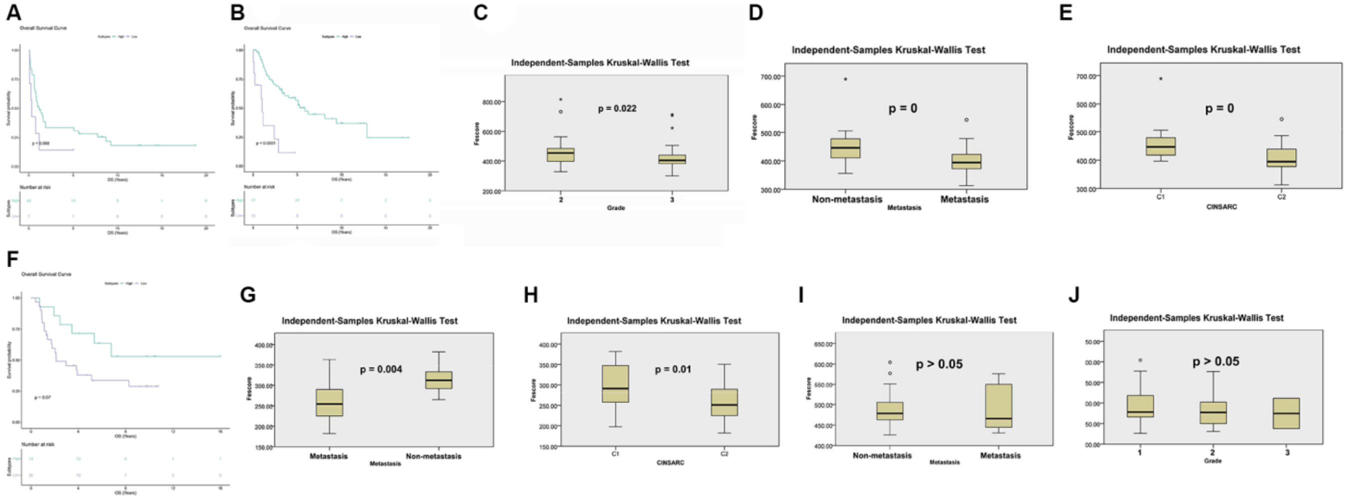


**Supplementary Figure 8. Validation of Fescore in different subtypes of STS.** (**A**) The survival plot between high and low Fescore group in UDS. (**B**, **C**) The survival plot and grading differences between high and low Fescore groups in LMS. (**D**, **E**) The metastasis and CINSARC index between high and low Fescore groups in SS. (**F**) The survival plot between high and low Fescore group in ES. (**G**, **H**) The metastasis and CINSARC index between high and low Fescore groups in FS. (**I**, **J**) The metastasis and Grading between high and low Fescore groups in FS.

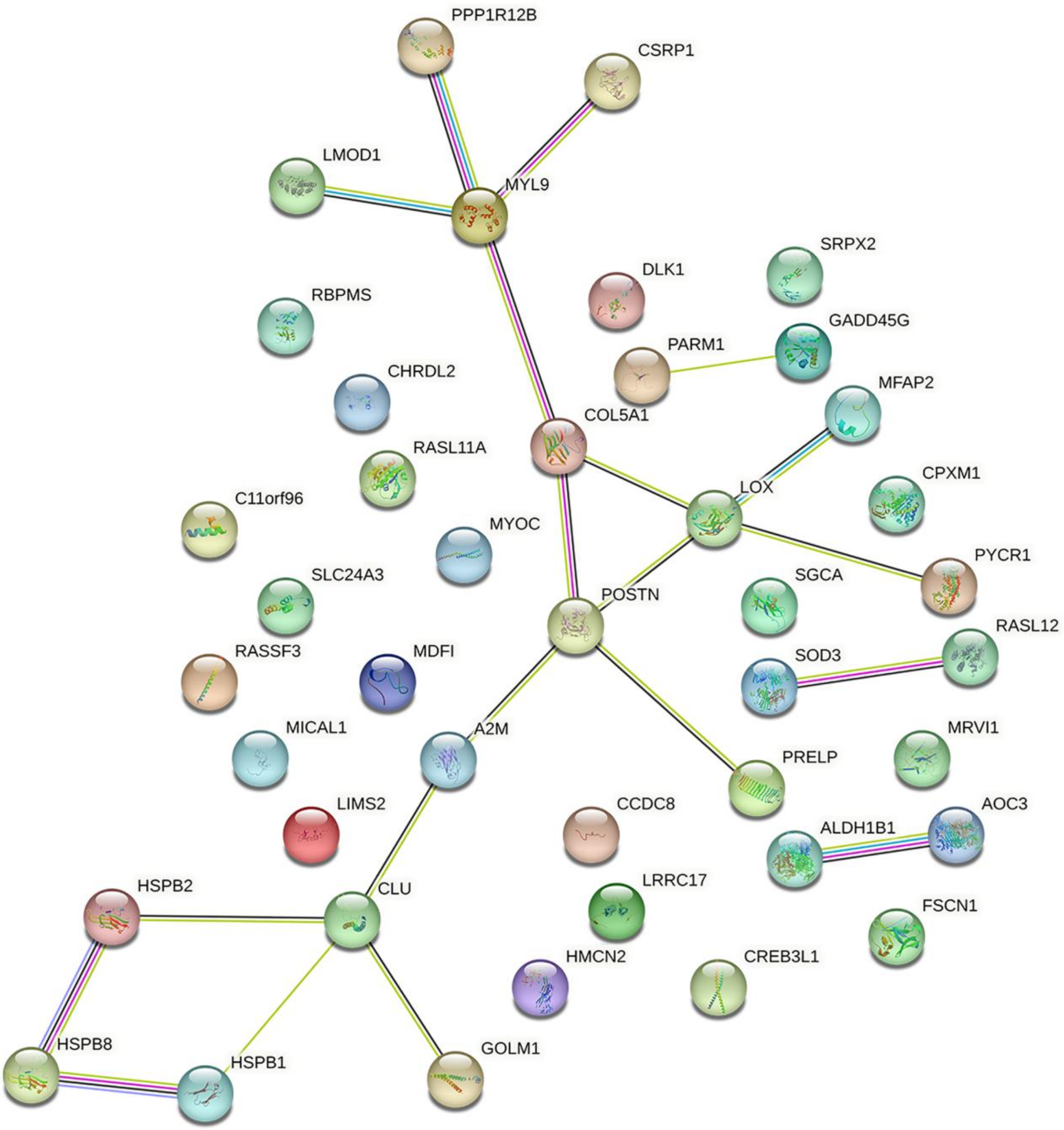


**Supplementary Figure 9. Protein-protein interaction analysis of genes that constructed Fescore.**
