# Supplementary material for: Molecular characterization of ferroptosis in soft tissue sarcoma constructs a prognostic and immunotherapeutic signature through experimental and bioinformatics analyses
Source: Aging (Albany NY). 2023 Oct 20;15(20):11412–47. doi: 10.18632/aging.205133 (PMC10637810; doi:10.18632/aging.205133)
Supplement: Supplementary Tables 1-7 and 9-11 [file aging-15-205133-s002.pdf]

SUPPLEMENTARY TABLES

Supplementary Table 1. Basic information of datasets included in this study.

Accession number/ Source	Platform	Number of patients	Number of samples used in this study	Research object	Survival data
GSE21122	Affymetrix Human Genome U133 Plus 2.0 Array	62	158	Human	OS
GSE35640	Affymetrix Human Genome U133 Plus 2.0 Array	65	56	Human	OS
GSE78220	Illumina RNAseq	28	28	Human	OS
IMvigor210	Illumina RNAseq	348	282	Human	OS
GSE119041	Affymetrix Human Genome U133 Plus 2.0 Array	50	50	Human	OS
GSE40021	Agilent-014850	58	58	Human	NA
GSE159848	Agilent-014850	50	50	Human	OS
GSE159847	Agilent-014850	87	87	Human	OS
GSE17618	Affymetrix Human Genome U133 Plus 2.0 Array	55	44	Human	OS
GSE71118	Affymetrix Human Genome U133 Plus 2.0 Array	312	58	Human	OS
TCGA: SARC	Illumina RNAseq	265	262	Human	OS
TCGA: LAML	Illumina RNAseq	151	132	Human	OS
TCGA: ACC	Illumina RNAseq	79	79	Human	OS
TCGA: CHOL	Illumina RNAseq	45	45	Human	OS
TCGA: BLCA	Illumina RNAseq	430	425	Human	OS
TCGA: BRCA	Illumina RNAseq	1217	1214	Human	OS
TCGA: CESC	Illumina RNAseq	309	296	Human	OS
TCGA: COAD	Illumina RNAseq	512	487	Human	OS
TCGA: UCEC	Illumina RNAseq	583	567	Human	OS
TCGA: ESCA	Illumina RNAseq	173	172	Human	OS
TCGA: GBM	Illumina RNAseq	173	167	Human	OS
TCGA: UCS	Illumina RNAseq	56	54	Human	OS
TCGA: HNSC	Illumina RNAseq	546	545	Human	OS
TCGA: KICH	Illumina RNAseq	89	87	Human	OS
TCGA: KIRC	Illumina RNAseq	607	602	Human	OS
TCGA: KIRP	Illumina RNAseq	321	318	Human	OS
TCGA: DLBC	Illumina RNAseq	48	47	Human	OS
TCGA: LIHC	Illumina RNAseq	424	418	Human	OS
TCGA: LGG	Illumina RNAseq	529	523	Human	OS
TCGA: LUAD	Illumina RNAseq	585	572	Human	OS
TCGA: LUSC	Illumina RNAseq	550	542	Human	OS
TCGA: SKCM	Illumina RNAseq	472	458	Human	OS
TCGA: MESO	Illumina RNAseq	86	84	Human	OS
TCGA: UVM	Illumina RNAseq	80	80	Human	OS
TCGA: OV	Illumina RNAseq	379	378	Human	OS
TCGA: PAAD	Illumina RNAseq	182	181	Human	OS
TCGA: PCPG	Illumina RNAseq	186	186	Human	OS
TCGA: PRAD	Illumina RNAseq	551	551	Human	OS
TCGA: READ	Illumina RNAseq	177	167	Human	OS
TCGA: STAD	Illumina RNAseq	407	381	Human	OS
TCGA: TGCT	Illumina RNAseq	156	139	Human	OS
TCGA: THYM	Illumina RNAseq	121	118	Human	OS
TCGA: THCA	Illumina RNAseq	568	567	Human	OS

Abbreviation: NA: Not applicable.

Supplementary Table 2. The list of ferroptosis regulators.

ACSL4
AKR1C1
AKR1C2
AKR1C3
ALOX15
ALOX5
ALOX12
ATP5MC3
CARS
CBS
CD44
CHAC1
CISD1
CS
DPP4
FANCD2
GCLC
GCLM
GLS2
GPX4
GSS
HMGCR
HSPB1
CRYAB
LPCAT3
MT1G
NCOA4
PTGS2
RPL8
SAT1
SLC7A11
FDFT1
TFRC
TP53
EMC2
AIFM2
PHKG2
HSBP1
ACO1
FTH1
STEAP3
NFS1
ACSL3
ACACA
PEBP1
ZEB1
SQLE

FADS2
NFE2L2
KEAP1
NQO1
NOX1
ABCC1
SLC1A5
GOT1
G6PD
PGD
IREB2
HMOX1
ACSF2

Supplementary Table 3. The sequences of siRNA for LOX (5′ to 3′).

siLOX-1:	GCAGGACTAGATGGAGCAA
siLOX-2:	TGGGACGAGTCAATCAACT
siLOX-3:	GATGGTGGATTACAATGGA

Supplementary Table 4. The prime sequences of all genes.

Genes	Prime sequences (5′–3′)
ACACA-F	AGGTGCCTAGAGGGTTGAAGA
ACACA-R	TCGGCCCTGCTTTACTAGGT
ACSF2-F	GCAGGCAGAGGATTTCAGTTC
ACSF2-R	TTCATGGAGGACGACCAAGG
AIFM2-F	TGATTCTCTGCACCGGCATC
AIFM2-R	GCTGGCTAGTCTGCTCTCAA
CBS-F	GACCAAGTTCCTGAGCGACA
CBS-R	CGGAGGATCTCGATGGTGTG
CD44-F	CTGCCGCTTTGCAGGTGTA
CD44-R	CATTGTGGGCAAGGTGCTATT
CRYAB-F	GGGGTCCTCACTGTGAATGG
CRYAB-R	TTCACGGGTGATGGGAATGG
FANCD2-F	ACATACCTCGACTCATTGTCAGT
FANCD2-R	TCGGAGGCTTGAAAGGACATC
GSS-F	GGTGAGCTATGCCCCATTCA
GSS-R	CACCAGAGCACTGGGCAAT
HMGCR-F	TTCGGTGGCCTCTAGTGAGA
HMGCR-R	GATGGGAGGCCACAAAGAGG
HSPB1-F	GCTTCACGCGGAAATACACG
HSPB1-R	GTGATCTCGTTGGACTGCGT
NCOA4-F	GAGGTGTAGTGATGCACGGAG
NCOA4-R	GACGGCTTATGCAACTGTGAA
NFE2L2-F	TCAGCGACGGAAAGAGTATGA

NFE2L2-R	CCACTGGTTTCTGACTGGATGT
NQO1-F	GACCTTGTGATATTCCAGAGTAAGA
NQO1-R	CCAGGCGTTTCTTCCATCCT
RPL8-F	TGTGATCCGTGGACAGAGGA
RPL8-R	GATCCCGGAAGACCACCTTG
SLC1A5-F	CACGTCCCACCCAGAGAAAC
SLC1A5-R	TGTCCGAAAGCTGGGAGTTC
SQLE-F	GTGCTGGTGTTCCTCTCGCT
SQLE-R	ATTGGTTCCTTTTCTGCGCCT
LOX-F	GGATACGGCACTGGCTACTT
LOX-R	TCTGGGTGTTGGCATCAAGC
GAPDH-F	GCACCGTCAAGGCTGAGAAC
GAPDH-R	TGGTGAAGACGCCAGTGGA

Supplementary Table 5. Functional enrichment analysis of differentially expressed ferroptosis regulators between normal and tumor tissues.

ID	Description	*P*-value	*p*.adjust	*q*-value	geneID	Count
GO:0006979	response to oxidative stress	5.5E-05	0.012817	0.004632	CRYAB/NQO1/HSPB1	3
GO:0045540	regulation of cholesterol biosynthetic process	2.14E-06	0.000601	0.000344	ACACA/HMGCR/SQLE	3
GO:0106118	regulation of sterol biosynthetic process	2.14E-06	0.000601	0.000344	ACACA/HMGCR/SQLE	3
GO:0090181	regulation of cholesterol metabolic process	4.32E-06	0.000742	0.000424	ACACA/HMGCR/SQLE	3
GO:0006695	cholesterol biosynthetic process	7.32E-06	0.000742	0.000424	ACACA/HMGCR/SQLE	3
GO:1902653	secondary alcohol biosynthetic process	7.62E-06	0.000742	0.000424	ACACA/HMGCR/SQLE	3
GO:0016126	sterol biosynthetic process	9.24E-06	0.000742	0.000424	ACACA/HMGCR/SQLE	3
GO:1902930	regulation of alcohol biosynthetic process	9.24E-06	0.000742	0.000424	ACACA/HMGCR/SQLE	3
GO:0050810	regulation of steroid biosynthetic process	1.49E-05	0.001048	0.000599	ACACA/HMGCR/SQLE	3
GO:0019218	regulation of steroid metabolic process	3.32E-05	0.001965	0.001123	ACACA/HMGCR/SQLE	3
GO:0006750	glutathione biosynthetic process	3.5E-05	0.001965	0.001123	GSS/NFE2L2	2
GO:0019184	nonribosomal peptide biosynthetic process	4.39E-05	0.002245	0.001282	GSS/NFE2L2	2
GO:0008203	cholesterol metabolic process	5.85E-05	0.002741	0.001566	ACACA/HMGCR/SQLE	3
GO:1902652	secondary alcohol metabolic process	6.45E-05	0.00279	0.001594	ACACA/HMGCR/SQLE	3
GO:0016125	sterol metabolic process	7.91E-05	0.003176	0.001814	ACACA/HMGCR/SQLE	3
GO:0046165	alcohol biosynthetic process	9.26E-05	0.003468	0.001981	ACACA/HMGCR/SQLE	3
GO:0044272	sulfur compound biosynthetic process	0.000122	0.004167	0.00238	GSS/ACACA/NFE2L2	3
GO:0006694	steroid biosynthetic process	0.00013	0.004167	0.00238	ACACA/HMGCR/SQLE	3
GO:0046890	regulation of lipid biosynthetic process	0.000133	0.004167	0.00238	ACACA/HMGCR/SQLE	3
GO:0010664	negative regulation of striated muscle cell apoptotic process	0.000209	0.006193	0.003538	HMGCR/NFE2L2	2
GO:0042398	cellular modified amino acid biosynthetic process	0.000312	0.008767	0.005008	GSS/NFE2L2	2

GO:1901617	organic hydroxy compound biosynthetic process	0.000337	0.009007	0.005145	ACACA/HMGCR/SQLE	3
GO:0006749	glutathione metabolic process	0.000392	0.009717	0.005551	GSS/NFE2L2	2
GO:0010665	regulation of cardiac muscle cell apoptotic process	0.000406	0.009717	0.005551	HMGCR/NFE2L2	2
GO:0010656	negative regulation of muscle cell apoptotic process	0.000435	0.009717	0.005551	HMGCR/NFE2L2	2
GO:0010662	regulation of striated muscle cell apoptotic process	0.000435	0.009717	0.005551	HMGCR/NFE2L2	2
GO:0010659	cardiac muscle cell apoptotic process	0.00045	0.009717	0.005551	HMGCR/NFE2L2	2
GO:0010658	striated muscle cell apoptotic process	0.00048	0.009991	0.005707	HMGCR/NFE2L2	2
GO:0007568	aging	0.000552	0.011088	0.006334	GSS/HMGCR/NFE2L2	3
GO:0051188	cofactor biosynthetic process	0.000578	0.011199	0.006398	GSS/ACACA/NFE2L2	3
GO:0008202	steroid metabolic process	0.000604	0.011317	0.006465	ACACA/HMGCR/SQLE	3
GO:0006066	alcohol metabolic process	0.000797	0.014442	0.00825	ACACA/HMGCR/SQLE	3
GO:0006790	sulfur compound metabolic process	0.000849	0.014902	0.008513	GSS/ACACA/NFE2L2	3

Supplementary Table 6. Enriched pathways in Fe cluster A.

NAME	ES	NES	NOM *p*-val	FDR *q*-val
HALLMARK_MYOGENESIS	0.634332	3.307229	0	0
HALLMARK_OXIDATIVE_PHOSPHORYLATION	0.604331	3.160266	0	0
HALLMARK_ADIPOGENESIS	0.479207	2.496	0	0
HALLMARK_INTERFERON_ALPHA_RESPONSE	0.503841	2.335555	0	0
HALLMARK_FATTY_ACID_METABOLISM	0.446815	2.273059	0	0
HALLMARK_REACTIVE_OXYGEN_SPECIES_PATHWAY	0.560496	2.267417	0	0
HALLMARK_BILE_ACID_METABOLISM	0.445523	2.114106	0	1.14E-04
HALLMARK_ESTROGEN_RESPONSE_LATE	0.389252	2.058489	0	3.29E-04
HALLMARK_UV_RESPONSE_UP	0.398036	2.016938	0	2.93E-04
HALLMARK_HEME_METABOLISM	0.357601	1.886102	0	8.47E-04
HALLMARK_INTERFERON_GAMMA_RESPONSE	0.355599	1.861583	0	8.33E-04
HALLMARK_KRAS_SIGNALING_DN	0.349546	1.806281	0	0.00131
HALLMARK_XENOBIOTIC_METABOLISM	0.335465	1.736298	0.0020408	0.002037
HALLMARK_ESTROGEN_RESPONSE_EARLY	0.321899	1.661753	0	0.003715
HALLMARK_PEROXISOME	0.35326	1.639113	0.001996	0.004375
HALLMARK_APICAL_JUNCTION	0.311443	1.632621	0	0.00443
HALLMARK_COAGULATION	0.304079	1.484184	0.0105042	0.018053
HALLMARK_CHOLESTEROL_HOMEOSTASIS	0.331957	1.471765	0.0347648	0.01924
HALLMARK_P53_PATHWAY	0.272602	1.404251	0.0217822	0.033998
HALLMARK_SPERMATOGENESIS	0.228275	1.128314	0.246124	0.227237

Supplementary Table 7. Enriched pathways in Fe cluster B.

NAME	ES	NES	NOM *p*-val	FDR *q*-val
HALLMARK_MYC_TARGETS_V2	−0.71611	−3.08285	0	0
HALLMARK_TGF_BETA_SIGNALING	−0.68269	−2.83247	0	0
HALLMARK_UNFOLDED_PROTEIN_RESPONSE	−0.55997	−2.68873	0	0
HALLMARK_MYC_TARGETS_V1	−0.5152	−2.67385	0	0
HALLMARK_PROTEIN_SECRETION	−0.55427	−2.59826	0	0
HALLMARK_EPITHELIAL_MESENCHYMAL_TRANSITION	−0.48943	−2.56332	0	0
HALLMARK_G2M_CHECKPOINT	−0.4862	−2.55363	0	0
HALLMARK_UV_RESPONSE_DN	−0.45711	−2.2864	0	0
HALLMARK_KRAS_SIGNALING_UP	−0.42344	−2.21573	0	0
HALLMARK_NOTCH_SIGNALING	−0.59168	−2.17589	0	0
HALLMARK_MITOTIC_SPINDLE	−0.4068	−2.15244	0	0
HALLMARK_WNT_BETA_CATENIN_SIGNALING	−0.54391	−2.14052	0	0
HALLMARK_MTORC1_SIGNALING	−0.4043	−2.11334	0	0
HALLMARK_ANGIOGENESIS	−0.55215	−2.08895	0	0
HALLMARK_E2F_TARGETS	−0.39287	−2.08114	0	0
HALLMARK_GLYCOLYSIS	−0.38931	−2.03895	0	0
HALLMARK_INFLAMMATORY_RESPONSE	−0.37657	−1.96033	0	0.00E+00
HALLMARK_TNFA_SIGNALING_VIA_NFKB	−0.34199	−1.82698	0	2.39E-04
HALLMARK_ALLOGRAFT_REJECTION	−0.33756	−1.7869	0.0020534	0.000825
HALLMARK_HEDGEHOG_SIGNALING	−0.47323	−1.76656	0.0094162	0.001076
HALLMARK_ANDROGEN_RESPONSE	−0.36871	−1.71922	0.0020747	0.001525
HALLMARK_COMPLEMENT	−0.3241	−1.7179	0	0.001456
HALLMARK_IL2_STAT5_SIGNALING	−0.32191	−1.70063	0	0.001571
HALLMARK_HYPOXIA	−0.30643	−1.62027	0.0019342	0.00444
HALLMARK_APOPTOSIS	−0.29261	−1.49237	0.001992	0.013702
HALLMARK_APICAL_SURFACE	−0.35631	−1.40262	0.0677656	0.031103
HALLMARK_DNA_REPAIR	−0.27639	−1.39083	0.0291971	0.03286
HALLMARK_IL6_JAK_STAT3_SIGNALING	−0.29632	−1.37348	0.0384615	0.037574

Supplementary Table 9. Functional enrichment analysis of DEGs between two ferroptosis modification patterns.

ID	Description	*p*-value	*p*.adjust	*q*-value	geneID	Count
GO:0005201	extracellular matrix structural constituent	8.44286E-12	3.05E-09	2.81E-09	POSTN/COL1A1/COL5A1/COL12A1/ SBSPON/DCN/FBLN2/NPNT/ HMCN2/MXRA5/SRPX2/LUM/ MFAP2/PCOLCE/PRELP	15
GO:0008307	structural constituent of muscle	4.38733E-09	7.92E-07	7.3E-07	MYL9/CSRP1/SYNM/PDLIM3/ MYH11/TPM1/TPM2/MYOM1	8
GO:0003779	actin binding	1.51403E-07	1.82E-05	1.68E-05	CAP2/SORBS1/ADD3/CNN1/SYNPO2/ FLNA/LMOD1/PDLIM3/MYH11/MYLK/ MICAL1/FSCN1/TAGLN/TMOD1/ TPM1/TPM2/MYOM1	17
GO:0051015	actin filament binding	7.37154E-06	0.000602	0.000555	ADD3/FLNA/MYH11/MICAL1/FSCN1/ TAGLN/TMOD1/TPM1/TPM2/MYOM1	10

GO:0016641	oxidoreductase activity, acting on the CH-NH2 group of donors, oxygen as acceptor	8.33687E-06	0.000602	0.000555	LOX/MAOA/MAOB/AOC3	4
GO:0016638	oxidoreductase activity, acting on the CH-NH2 group of donors	2.1615E-05	0.001301	0.001198	LOX/MAOA/MAOB/AOC3	4
GO:0048407	platelet-derived growth factor binding	9.36937E-05	0.004832	0.004452	COL1A1/COL5A1/PDGFRA	3

Supplementary Table 10. Cmap analysis for DEGs.

Cmap name	Mean	*n*	Enrichment	*p*	Specificity	Percent non-null
Doxylamine	−0.655	5	−0.888	0.00006	0	100
Sulfadimethoxine	−0.399	5	−0.851	0.00022	0	60
Carteolol	−0.266	4	−0.837	0.00123	0	50
3-acetamidocoumarin	−0.352	4	−0.837	0.00125	0.026	50
PHA-00851261E	0.421	8	0.627	0.00154	0	75
Sulfaquinoxaline	−0.646	3	−0.899	0.00192	0.0062	100
AH-6809	−0.707	2	−0.961	0.0033	0.0056	100
Harmine	0.31	4	0.796	0.00342	0.0076	50
Karakoline	−0.371	6	−0.653	0.00475	0	50
Difenidol	0.455	3	0.863	0.00481	0.0152	66
Sulfadiazine	−0.528	5	−0.684	0.00717	0.0263	80
Isoniazid	−0.388	5	−0.672	0.00885	0.0497	60
Cefotiam	−0.282	4	−0.741	0.00891	0.0065	50
Hydrocortisone	0.382	3	0.834	0.00915	0	66
Etiocholanolone	−0.402	6	−0.612	0.01124	0.1948	66
Mafenide	−0.291	5	−0.654	0.0122	0.018	60
STOCK1N-35874	0.649	2	0.92	0.01272	0.0435	100
Proscillaridin	0.491	3	0.813	0.01302	0.0842	66
Withaferin A	0.624	4	0.71	0.01452	0.1947	75
16-phenyltetranorprostaglandin E2	−0.535	4	−0.708	0.0151	0.0298	75
Sitosterol	0.261	4	0.7	0.01667	0.0172	50
PF-00539745-00	−0.384	3	−0.798	0.01703	0.0815	66
Puromycin	0.474	4	0.695	0.01818	0.2472	75
Ticarcillin	0.366	3	0.789	0.01889	0.0133	66
Flunarizine	0.35	4	0.691	0.01898	0.034	50
Gliquidone	0.519	4	0.69	0.01932	0	75
Cyproterone	0.328	4	0.677	0.02367	0.024	50
Alverine	−0.354	4	−0.672	0.02586	0	75
Clenbuterol	−0.407	5	−0.598	0.03004	0.0397	60
Oxybenzone	−0.369	4	−0.657	0.03191	0.0986	50
Practolol	0.258	4	0.649	0.03599	0.0259	50
Abamectin	−0.493	4	−0.646	0.0375	0.0617	75

Diazoxide	−0.356	5	−0.574	0.04189	0.061	60
PHA-00767505E	0.433	4	0.636	0.04307	0.0387	75
Harmalol	−0.479	3	−0.724	0.04333	0.052	66
Sparteine	0.481	4	0.635	0.04345	0.0072	75
Econazole	0.242	4	0.632	0.04552	0.1949	75
Nialamide	0.429	4	0.63	0.04677	0.036	75
5255229	0.34	2	0.847	0.0472	0.0474	50
Indoprofen	−0.314	4	−0.626	0.04852	0.1333	50
Colecalciferol	−0.409	4	−0.625	0.04886	0.0272	75
Cinnarizine	0.549	4	0.625	0.04933	0.0584	75

Supplementary Table 11. Full list of 43 prognostic DEGs.

	cox_HR
LIMS2	0.382144
AOC3	0.481967
SOD3	0.495754
C11orf96	0.543024
HMCN2	0.543768
A2M	0.546305
GADD45G	0.554395
HSPB8	0.573024
CLU	0.573242
MYOC	0.582298
RASL11A	0.582661
CSRP1	0.59421
MRVI1	0.618517
PARM1	0.622781
RASSF3	0.629226
SLC24A3	0.634694
PPP1R12B	0.634896
CHRDL2	0.641911
HSPB2	0.642656
RBPMS	0.645255
RASL12	0.649648
SGCA	0.649931
MICAL1	0.652491
LMOD1	0.65268
HMGN2P19	0.65459
MYL9	0.663697
HSPB1	0.663706
ALDH1B1	0.666453
PRELP	0.667319
DLK1	1.499914
POSTN	1.530762
COL5A1	1.53084
SRPX2	1.543947

FSCN1	1.587907
CREB3L1	1.594449
CCDC8	1.631176
GOLM1	1.63245
CPXM1	1.658367
MDFI	1.665139
PYCR1	1.732861
LOX	1.819126
MFAP2	1.903821
LRRC17	2.177627
